# Design choices: Empirical recommendations for designing two-dimensional finger-tracking experiments

**DOI:** 10.3758/s13428-020-01409-0

**Published:** 2020-05-15

**Authors:** Robert Wirth, Anna Foerster, Wilfried Kunde, Roland Pfister

**Affiliations:** Department of Psychology, Julius-Maximilians-University of Würzburg, Röntgenring 11, 97070 Würzburg, Germany

**Keywords:** Movement tracking, Experimental design, Simon task, Measures

## Abstract

The continuous tracking of mouse or finger movements has become an increasingly popular research method for investigating cognitive and motivational processes such as decision-making, action-planning, and executive functions. In the present paper, we evaluate and discuss how apparently trivial design choices of researchers may impact participants’ behavior and, consequently, a study’s results. We first provide a thorough comparison of mouse- and finger-tracking setups on the basis of a Simon task. We then vary a comprehensive set of design factors, including spatial layout, movement extent, time of stimulus onset, size of the target areas, and hit detection in a finger-tracking variant of this task. We explore the impact of these variations on a broad spectrum of movement parameters that are typically used to describe movement trajectories. Based on our findings, we suggest several recommendations for best practice that avoid some of the pitfalls of the methodology. Keeping these recommendations in mind will allow for informed decisions when planning and conducting future tracking experiments.

## Introduction

The analysis of continuous-movement trajectories has become an increasingly popular method in recent years for psychological research. Movement tracking not only allows the analysis of the final outcome of a decision process (i.e., in terms of choice frequencies or response times as in common button-press tasks), but also offers parameters that capitalize on the decision process itself, and have often been claimed to show how it unfolds over time (Freeman, Dale, & Farmer, 2011; McKinstry, Dale, & Spivey, 2008; Song & Nakayama, 2009; Spivey, Grosjean, & Knoblich, 2005). There are several variants of this approach, for example movement tracking in a three-dimensional space (Buetti, Juan, Rinck, & Kerzel, 2012; Erb, Moher, Sobel, & Song, 2016; Erb, Moher, Song, & Sobel, 2018; Song & Nakayama, 2008) or the projection of a three-dimensional movement onto a two-dimensional plane (using a Nintendo Wiimote; Duran, Dale, & McNamara, 2010). There are setups in which participants approach only one target (Schween & Hegele, 2017), decide between multiple discrete response options (e.g., Deubel & Schneider, 2003; Mahon, Bendžiūtė, Hesse, & Hunt, 2020), or make their choice on a continuous scale (Debats & Heuer, 2018). But the currently most prominent approach employs a movement in the 2D plane in which participants decide between two discrete options by moving a cursor to one of two target areas.

Cursor movements are typically performed via the computer mouse, though recent studies have also used finger-tracking on a touchscreen device in similar tasks (e.g., Wirth, Kunde, & Pfister, 2019). In typical setups for mouse- or finger-tracking, participants start with the mouse cursor or their finger at the bottom center of the screen, and they choose an option by moving the cursor to one of two target areas located in the upper corners of the screen. Even within this apparently simple setup, there are a range of design choices that researchers must make and that can lead to consequences for the behavior in question (e.g., Kieslich, Schoemann, Grage, Hepp, & Scherbaum, 2020). For example, parameters such as the size of the target areas and the distance of the target areas from the starting position can result in fundamentally different movements, with large target areas at a short distance requiring less spatially accurate movements than with small target areas that are placed far away (Fitts, 1954).

Still, one might argue that as long as these design choices are kept constant across conditions, these choices will be trivial. However, with the present study, we want to sensitize researchers to such apparently trivial design choices, which are, as we will show here, actually not that trivial and need careful consideration when designing a movement-tracking experiment. Further, we believe that considering these design choices is also important when it comes to interpreting such tracking experiments, as well as when conducting replications or meta-analyses.

What all tracking setups have in common is that the ensuing movement trajectory is continuously logged and thereby offers a plethora of measures that can be analyzed (Hehman, Stolier, & Freeman, 2015). The “standard” measure of how long it took to complete a trial (response time in classical discrete response tasks) can now be further differentiated into the time it took to initiate and execute the movement. This may allow for a coarse distinction of cognitive processes that are in charge of movement planning from those that govern movement execution. Spatial parameters, such as the deviation from an optimal movement line, can indicate attraction towards one or the other response option, whereas directional changes during movement execution can reflect the continuous decision process as it unfolds during response execution. Via a combination of temporal and spatial parameters, speed and acceleration profiles of each movement can be derived and analyzed. But again, this wide spectrum of measures provides researchers with a high degree of freedom, and depending on the research question, allows for a tailored selection of interesting measures, in which each measure can have its own advantages when it comes to its theoretical and/or practical implication. With this wide spectrum, it is not surprising that researchers use vastly different measures to operationalize the very same theoretical concept (e.g., describing the completion of a decision process when a movement is initiated, Greenwood & Spivey, 2014, or alternatively only when the peak movement velocity is reached, Barca & Pezzulo, 2015). In addition to assessing the impact of different design choices, the second goal of this research is thus to give an overview of the most frequently used measures in movement tracking, and to assess their effectiveness for capturing differences between experimental conditions.

The following five experiments offer a fine-grained exploratory empirical contribution to a rising field of psychological research. We manipulated several design parameters and tested how they affect participants’ movement performance across a wide spectrum of dependent variables (DVs, see below for more details). Even though not every design choice allows for a clear prediction of whether and how it will affect each DV that we derive from these movements, we will analyze every movement parameter in every experiment to provide a full picture of the empirical data. Unless stated otherwise, take these analyses (especially the full results, www.osf.io/am6yp) as a helpful exploratory overview to support researchers in estimating how their design choices may affect their DVs of interest.

## Experiment 1: Manipulation of input device

### Introduction

In Experiment 1, we tested how the input device that is used for movement tracking might influence movement trajectories (Moher & Song, 2019). Following recent trends, we compared two setups that use a computer mouse to measure continuous movements (e.g., Jusyte et al., 2017; Pfister, Wirth, Schwarz, Steinhauser, & Kunde, 2016; Scherbaum, Dshemuchadse, Fischer, & Goschke, 2010; Tabatabaeian, Dale, & Duran, 2015) as well as a setup that takes advantage of the touchscreen of a tablet computer (e.g., Kunde, Schmidts, Wirth, & Herbort, 2017; Wirth, Pfister, Foerster, Huestegge, & Kunde, 2016).

For the two mouse-operated setups, we compared the “MouseTracker,” as a frequently used, ready-made program (Freeman & Ambady, 2010), with a similar custom-built program that also uses a computer mouse (“eTracker”). Further, we aimed to parallelize these programs with the one that uses a touchscreen for input (“iTracker”). We decided to test two mouse-operated setups to gauge the impact of several limitations that necessarily arise when using ready-made software. For the MouseTracker, these limitations especially pertain to the limited choices within the setup and options to provide feedback to the participants, as we will describe below.^1^ All participants performed the same task on all setups in counterbalanced order, which allowed us to compare how the choice of input method may influence movement trajectories.

We designed a simple Simon task (Simon, 1990) in which one of two target areas changed color on each trial. Color identity, not color location, indicated whether an upward left or an upward right movement was required. As such, target position and movement direction could be either compatible (i.e., movement towards the stimulus color) or incompatible (i.e., movement to the opposite side of the stimulus color). Performance is usually worse in incompatible relative to compatible trials. This Simon effect is among the most robust and most frequently observed phenomena in cognitive psychology (e.g., Hommel, 2011). It should thus emerge with any setup, though perhaps to varying degrees and in different dependent measures, and we were interested in exactly these varying degrees depending on the setup.

The Simon effect is further subject to a variation with trial sequence. Typically, the Simon effect is smaller after incongruent rather than congruent trials, which is known as sequential adaptation effect. The reasons for this sequential adaptation effect are disputed (e.g. Hommel, Proctor, & Vu, 2004; Egner, 2007), but the phenomenon as such seems equally robust as the Simon effect itself. Therefore, we also analyzed the data according to congruency sequence, to get an idea of how the type of input device influences this effect.

Regarding the quantification of movement trajectories, the available literature offers a diverse set of dependent variables (DVs), but there is currently no consensus on which variables to use. Ideally, researchers determine a priori which DVs to use (and we will come back to this point in the discussion). In the current experiments, we specifically aimed at giving a systematic overview of which markers best differentiate between the experimental conditions in the present setup, and we therefore analyzed all of the most common temporal and spatial DVs (full results for each individual DV are reported in the Supplementary Material online, www.osf.io/am6yp). Even though different research questions may require different variables, this approach will allow for informed choices between conceptually similar measures for different study designs. Finally, we not only took objective and implicit measures into account to differentiate between the setups, but we also considered explicit, subjective assessments by running a questionnaire after the experiment, in which participants judged the setups on several scales.

### Methods

#### Participants

Thirty-six participants were recruited (mean age = 26.8 years, *SD* = 5.3, 9 male, 3 left-handed) and received either course credit or €5 monetary compensation. This sample size was based on the minimum number of participants required to counterbalance the order of experimental setups and stimulus–response mappings across subjects. All participants gave informed consent, were naïve to the purpose of the experiment and were debriefed after the session.

#### Apparatuses

For a thorough comparison of the available mouse-tracker methods, we built identical Simon tasks within the *MouseTracker* (www.mousetracker.org, e.g., Freeman & Ambady, 2010; Faulkenberry, 2014), a version built in E-Prime 2.0 (*eTracker*, www.roland-pfister.net, e.g., Pfister, Janczyk, Wirth, Dignath, & Kunde, 2014; Wirth, Pfister, Janczyk, & Kunde, 2015), and on an iPad 2 (*iTracker*, e.g., Dignath, Wirth, Kühnhausen, Gawrilow, Kunde, & Kiesel, 2020; Wirth, Dignath, Pfister, Kunde, & Eder, 2016; Wirth, Pfister, & Kunde, 2016) in portrait mode (i.e., the shorter side of the device is oriented horizontally). While the computer setups were operated with a computer mouse, the iPad required input via a finger on its touchscreen. Clicking the mouse was equated with touching the screen or lifting the finger from the screen. Cursor acceleration on the computers was turned off and cursor speed was turned down so that hand and cursor moved at a comparable speed (cursor settings in Windows were at 30% of the maximum possible speed, with gain disabled); on the iPad, finger and cursor movements were inherently identical. Participants worked on the same experimental task on all three setups; the order of the setups was counterbalanced between participants. The experimental computers had 17” displays running at a display resolution of 1024 × 768 px, whereas the iPads had a screen size of 9.7” running at the same display resolution but were operated at a shorter viewing distance. All experiments were run in full screen mode.

#### Stimuli and procedure

Participants were confronted with a starting area (black circle 60 px in diameter) at the bottom of the screen against a white background. Upon clicking or touching the screen within the starting area, the starting area disappeared, and two target areas appeared (two circles 60 px in diameter upwards to the left and upwards to the right of the starting area). The MouseTracker does not allow for circular starting and target areas, even though circular areas seem reasonable especially for the starting area, because otherwise the angle at which the movement is initiated would confound the time at which movement initiation is determined (usually when leaving the starting area)^2^. Therefore, we used square boxes of 60 px side length in the MouseTracker-version and addressed this inconsistency during data preprocessing by using virtual circular starting and target areas for the computation of our dependent variables.

The centers of the target areas were 600 px above the center of the starting area and displaced at 300 px to either the left or right. Consequently, straight lines from the center of the starting area to the centers of each target area would produce angles of 26.6° to each side relative to a vertical midline. Crucially, either the left or the right target area turned red or green, while the other target area was black. For half of the participants, red color prompted a movement to the left target area, and green color prompted a movement to the right target area. The other half received the opposite stimulus–response mapping for counterbalancing. Each participant had the same stimulus–response mapping throughout all setups. With this manipulation, movements could be stimulus–response-compatible (movement towards the colored target area) or -incompatible (movement away from the colored target area). If a target area was reached, either it had to be clicked or the finger had to be lifted within the target area to complete a trial.

From clicking/touching the starting area to clicking/lifting the finger from the target area, X- and Y-coordinates of the movement were sampled. The MouseTracker and iTracker sampled movement data at 60 Hz (although the iTracker logged the data more frequently at about 200 Hz), and the eTracker’s sample rate depended on the current CPU load and was therefore more variable, but overall comparable to the other devices. Based on these coordinate data, all dependent measures were computed (see *preprocessing*).

After correct responses, the next trial started instantly with the presentation of the starting area. After commission errors (“Fehler!”, German for “error!”), after response initiations slower than 1000 ms (“Zu langsam gestartet!”, “started too slowly!”), after movement executions slower than 1500 ms (“Zu langsam!”, “too slow!”), and after omission errors, i.e., prematurely lifting the finger from the touchscreen (“Nicht getroffen!”, “missed!”; only in the iTracker version), feedback was displayed in red font in the center of the screen for 1000 ms. The next trial could still be started immediately. With the MouseTracker, we were not able to make all types of feedback work; it only allowed us to show feedback for either slow initiation or slow execution. Therefore, we decided to omit speed-related feedback altogether with the MouseTracker and to only present feedback on commission errors. Nevertheless, we identified all remaining error types in our statistical analysis.

A block consisted of 100 trials in randomized order, with an equal number of compatible and incompatible trials, and an equal number of required left and right responses. Participants completed six blocks overall, two consecutive blocks per setup (AABBCC), with short, self-paced breaks between blocks.

#### Questionnaire

At the end of the experiment, participants completed a paper questionnaire (German version available at www.osf.io/am6yp). Each setup had to be rated on a nine-point scale with semantic labels for the outermost categories (in brackets), concerning their accessibility (i.e., the first trials, difficult–easy), two ratings regarding their overall experience in handling the devices (difficult–easy; unpleasant–pleasant), estimates of the movements (stuttering–smooth; exhausting–effortless), perceived control of the movements (little–full), and their error robustness (little–very). The devices were rated in the order that they were worked on during the experiment, so participants were asked for each question to rate “Part 1”, then “Part 2”, and then “Part 3” before proceeding to the next question. Finally, they answered which setup they would prefer if they had to do the experiment again.

### Results

#### Overall analysis strategy

**Fig. 1:**
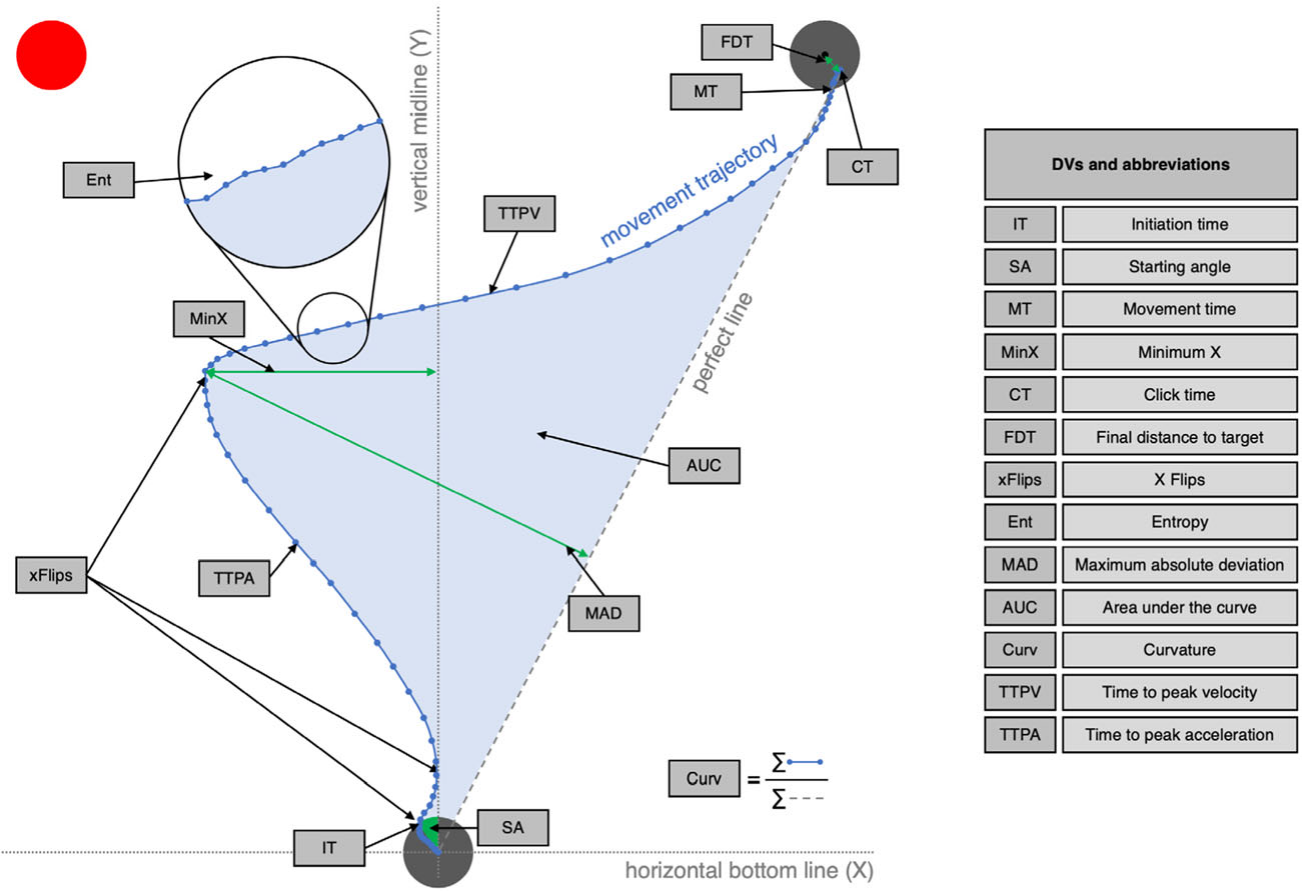
Overview of possible dependent variables when analyzing movement trajectory data. The circular area at the bottom represents the starting area; the two on the top left and top right represent the target areas. A trial was started by clicking or touching the starting area, making the stimulus appear. In this case, the left target area turned red, with red color indicating a movement to the right; therefore, this would constitute an incompatible trial. What is depicted here is the actual data for a single trial of the experiment, the blue dots representing the cursor/finger position over time. This exemplary movement was initiated in the wrong direction. The distance between the dots lets us estimate the speed profile of the movement, with longer distances between dots indicating faster movements. A trial was completed when a target area was clicked or the finger was lifted from the screen within a target area

**Fig. 2:**
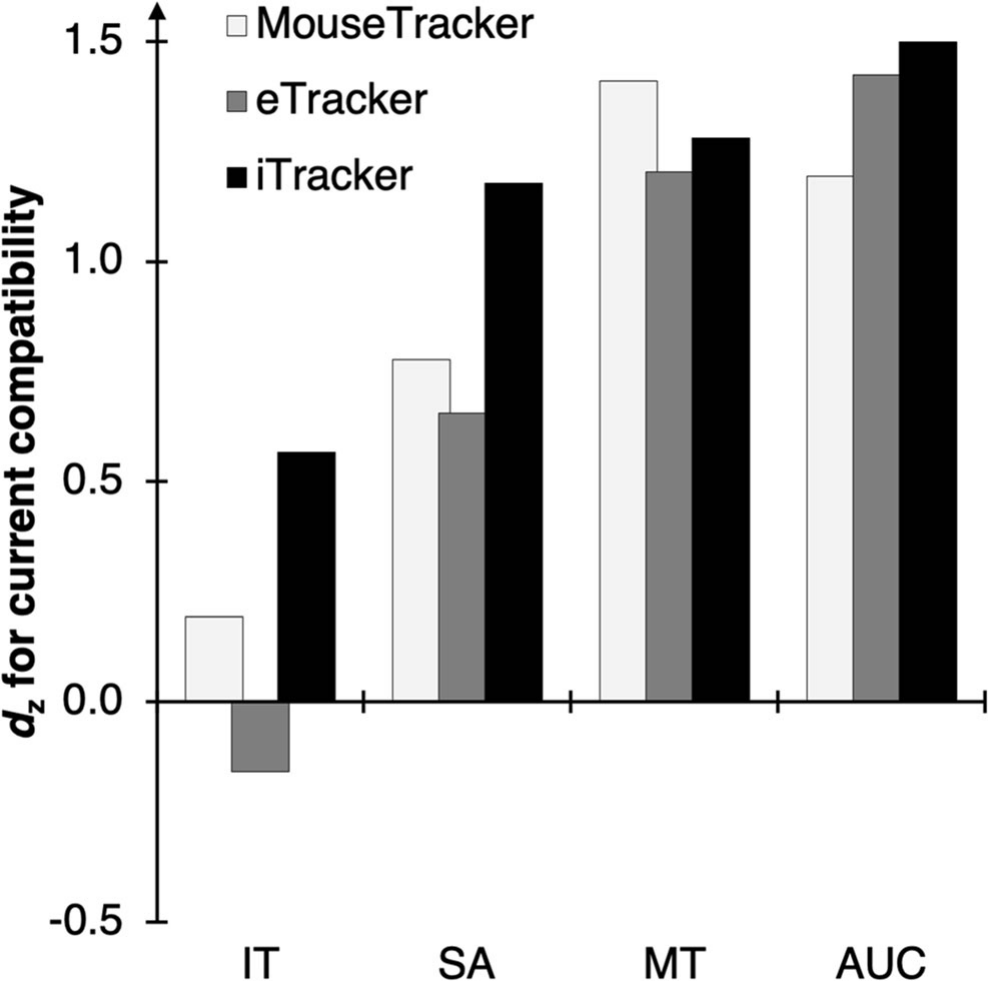
Main results of Experiment 1. Standardized effect sizes *d*_z_ for the effect of current compatibility (computed as current incompatible minus current compatible) for each of the computed DVs (*X*-axis) and setups (columns)

In the following sections, we first describe data selection and preprocessing, including a detailed list of all computed statistics (see Fig. 1 for a graphical summary). An overview of the most relevant results is provided in Fig. 2. Standardized effect sizes for paired comparisons are computed as Cohen’s *d*_*z*_ = $\frac{t}{\sqrt{n}}$ .

#### Data selection

For the following analyses, we omitted trials in which participants produced commission errors (5.3%) or omissions (7.5%). Error and omission rates were analyzed via linear mixed-effects models using the lme4 package version 1.1-21 of the R software environment. For all analyses we report the outcome of appropriate model comparisons for a model including the effect of interest to the corresponding null model. More errors were committed in the MouseTracker (8.7%) than in the eTracker (3.6%) or in the iTracker (3.7%), *Χ*^*2*^(1) ≥ 169.54, *p*s < .001, with no difference between the latter setups, *Χ*^*2*^(1) = 0.05, *p* = .821, and more omissions in the iTracker (12.4%)^3^ than in the MouseTracker (2.7%)^4^ or the eTracker (0.0%), with significant differences between all setups, *Χ*^*2*^(1) ≥ 196.44, *p*s < .001. To provide the most conservative comparison between the setups, the remaining data entered analyses unfiltered. Thereby, strong variations of any measure are not artificially narrowed via outlier elimination, but considered within the analyses.

#### Preprocessing and data analyses

First, the X- and Y-coordinate data of the MouseTracker were converted from its native logfiles via custom R scripts and restructured to fit the data format of the eTracker and iTracker, making it possible to run pooled analyses on all data. Further, this allowed for the use of virtual circular starting and target areas for the computation of our dependent variables, even though the MouseTracker only allows for rectangular areas. For all following computations, we defined the origin of the coordinate system to be located at the center of the home area. Positive values in the X-direction indicate movements to the right, and positive values in the Y-direction indicate upward movement.

The data from all setups was then analyzed via custom MATLAB scripts. Movements to the left were mirrored on the vertical midline. Based on the raw coordinate data, the following measures were computed for each movement (see Fig. 1)^5^:

- Initiation time (IT, in ms): Time interval between the click/touch of the starting area and leaving the starting area (criterion: Euclidian distance between the center of the starting area and the current cursor position is larger than 30 px)
- Starting angle (SA, in °): Angle of the movement when leaving the starting area relative to the vertical midline (with movements straight up producing an angle of 0°, positive values indicating movement directions towards the correct target area, and negative values indicating initial movements towards the opposite side. For reference, a straight line from start- to endpoint of the movement—see perfect line, Fig. 1—produced an angle of 26.6°)
- Movement time (MT, in ms): Time interval between leaving the starting area and entering a target area (criterion: Euclidian distance between center of the target area and current cursor position is smaller than 30 px)
- Minimum X (MinX, in px): Maximum deviation in the opposite direction of the correct target area on the *X*-axis
- Click time (CT, in ms): Time interval between entering the target area and clicking/lifting the finger
- Final distance to target (FDT, in px): Distance between the center of the target area and the final coordinate of the movement
- X flips (xFlips, as integer): Number of instances when the movement changes direction on the *X*-axis

Next, the data for the movement execution (during MT) was time-normalized to 101 steps, and based on this data, further parameters were extracted:^6^

- Entropy (Ent): Indicator of movement complexity, with larger values for more complex movements, based on Hehman et al. (2015)
- Maximum absolute deviation (MAD, in px): Maximum movement deviation from the perfect line (deviations towards the opposing target area were coded as positive values, deviations towards the nearest edge of the screen produced negative values)
- Area under the curve (AUC, in px^2^): Area between the actual movement and the perfect line (deviations towards the opposing target area were coded as positive values, deviations towards the nearest edge of the screen produced negative values)
- Curvature (Curv, as ratio): Ratio of the length of the actual movement and the length of the perfect line
- Time to peak velocity (TTPV, in % of movement): Time point with maximum movement speed
- Time to peak acceleration (TTPA, in % of movement): Time point with maximum movement acceleration

All these DVs extract a single metric from the continuous data that can be tested by common inferential statistics methods. They do not come with any additional prerequisites and allow for an easy-to-grasp overview of the data, and they should be robust against specific design choices (Scherbaum & Kieslich, 2018). Therefore, possible modulations of the Simon effect or its sequential adaptation by the setup should be especially meaningful. More advanced statistical methods have been proposed that are explicitly tailored to specific research questions in the context of continuous data (e.g., Joch, Döhring, Maurer, & Müller, 2019; Maldonado, Dunbar, & Chemla, 2019; Scherbaum, Dshemuchadse, Fischer, & Goschke, 2010), and we provide the raw data online to enable the application of these approaches (www.osf.io/am6yp).

All dependent measures were aggregated as the mean per participant and per condition and then analyzed via 2 × 2 × 3 analyses of variance (ANOVAs)^7^ with current compatibility (trial N compatible vs. incompatible), preceding compatibility (trial N-1 compatible vs. incompatible), and setup (MouseTracker vs. eTracker vs. iTracker) as within-subject factors. We refer to compatibility effects as the difference between currently compatible and incompatible trials (computed as trial N incompatible minus trial N compatible, see Fig. 2), to aftereffects as the difference between trials after compatible and after incompatible trials (computed as trial N-1 incompatible minus trial N-1 compatible) and to sequential adaptation effects as the modulation of compatibility effects by preceding compatibility (in the direction of smaller compatibility effects after an incompatible trial relative to after a compatible trial; Gratton, Coles, & Donchin, 1992).

Since we are not interested in the Simon effect or its sequential modulation per se, but how they might be modulated by the setup, we mainly focused on any effect including the factor setup. Main effects of current compatibility, preceding compatibility, or their interaction serve as a manipulation check, and descriptive means for the main effects are provided to give an estimate of the absolute values of the individual DVs. To keep the results frugal and accessible, we only scrutinized effects which include the factor setup in follow-up analyses via planned two-tailed *t* tests to compare which setup produced the largest Simon effect. Accordingly, in case of differences in sequential modulation, we tested for sequential adaptation within each setup via separate ANOVAs to see which setup produced a significant adaptation pattern.

For the same reasons, we report the full analysis of all DVs online (www.osf.io/am6yp). For the main text, we will focus on IT, SA, MT, and AUC, as they provide an overview of both temporal and spatial measures of both early and late stages of the movement (for a more elaborate reasoning for these DVs, as well as a correlation matrix of all DVs and a factor analysis akin to Incera, 2018, also see www.osf.io/am6yp). Temporal measures are of interest for the current setup because incompatibility of stimulus and response location is expected to prolong action planning, and the incompatible location of a stimulus should further attract the trajectory (relative to compatible trials; Buetti & Kerzel, 2008; Wirth, Foerster, Herbort, Kunde, & Pfister, 2018).

#### Initiation times

Data showed significantly faster response initiation for current compatible trials (249 ms) than for incompatible trials (254 ms), *F*(1, 35) = 8.36, *p* = .007, η_p_^2^ = .19, as well as faster response initiation after compatible trials (250 ms) than after incompatible trials (254 ms), *F*(1, 35) = 9.68, *p* = .004, η_p_^2^ = .22. Response initiation was slower in the iTracker (380 ms) relative to the eTracker (201 ms) and MouseTracker (174 ms), *F*(2, 34) = 89.90, *p* < .001, η_p_^2^ = .84, with significant differences between all setups, *t*s ≥ 4.70, *p*s < .001, *d*s ≥ 0.78. Compatibility effects differed between setups, *F*(2, 34) = 5.93, *p* = .006, η_p_^2^ = .26, with the iTracker producing significantly larger effects (∆ = 14 ms) compared to the eTracker (∆ = −1 ms) or the MouseTracker (∆ = 2 ms), *t*s ≥ 3.00, *p*s ≤ .005, *d*s ≥ 0.50, but no difference between the latter setups, *t*(35) = 1.19, *p* = .241, *d* = 0.20. Overall, sequential adaptation effects emerged, *F*(1, 35) = 7.41, *p* = .010, η_p_^2^ = .18, but these were further modulated by setup, *F*(2, 34) = 5.71, *p* = .007, η_p_^2^ = .25, showing that only the iTracker produced the sequential adaptation effect, *F*(1, 35) = 9.75, *p* = .004, η_p_^2^ = .22, and not the others, *F*s ≤ 2.41, *p*s ≥ .129. Aftereffects did not differ between setups, *F* < 1.

#### Starting angles

Data showed significantly steeper response initiation for current incompatible trials (−1.3°) than for compatible trials (4.8°), *F*(1, 35) = 46.77, *p* < .001, η_p_^2^ = .57, as well as steeper response initiation after compatible trials (1.3°) than after incompatible trials (2.2°), *F*(1, 35) = 4.78, *p* = .036, η_p_^2^ = .12. Response initiation was most direct in the iTracker (6.8°) relative to the eTracker (-3.2°) and MouseTracker (1.7°), *F*(2, 34) = 41.92, *p* < .001, η_p_^2^ = .71, with significant differences between all setups, *t*s ≥ 3.81, *p*s ≤ .001, *d*s ≥ 0.63. A significant three-way interaction, *F*(2, 34) = 8.64, *p* = .001, η_p_^2^ = .34, indicated that only the iTracker produced the expected sequential adaptation effect, *F*(1, 35) = 9.16, *p* = .005, η_p_^2^ = .21, whereas the MouseTracker did not, *F* < 1, and the eTracker produced a significant interaction, but in the opposite direction, *F*(1, 35) = 8.55, *p* = .006, η_p_^2^ = .20. No other effects were significant, *F*s < 1.

#### Movement times

Data showed significantly faster response execution for current compatible trials (438 ms) than for incompatible trials (488 ms), *F*(1, 35) = 111.27, *p* < .001, η_p_^2^ = .76, as well as faster response execution after incompatible trials (460 ms) than after compatible trials (467 ms), *F*(1, 35) = 9.11, *p* = .005, η_p_^2^ = .21. Response execution was fastest in the iTracker (419 ms) relative to the eTracker (481 ms) and MouseTracker (489 ms), *F*(2, 34) = 9.92, *p* < .001, η_p_^2^ = .37, with significant differences between the iTracker and both of the other two, *t*s ≥ 4.15, *p*s < .001, *d*s ≥ 0.69, but no difference between eTracker and MouseTracker, *t*(35) = 1.14, *p* = .263, *d* = 0.19. Sequential adaptation effects emerged, *F*(1, 35) = 69.15, *p* < .001, η_p_^2^ = .66. No other effects were significant, *F*s ≤ 2.96, *p*s ≥ .066.

#### Area under the curve

Movements showed significantly greater spatial deviations for current incompatible trials (61427 px^2^) than for compatible trials (36842 px^2^), *F*(1, 35) = 103.25, *p* < .001, η_p_^2^ = .75, as well as after compatible trials (51689 px^2^) relative to after incompatible trials (46580 px^2^), *F*(1, 35) = 28.25, *p* < .001, η_p_^2^ = .45. Overall deviation was smallest in the iTracker (23274 px^2^) relative to the eTracker (64271 px^2^) and MouseTracker (59860 px^2^), *F*(2, 34) = 77.00, *p* < .001, η_p_^2^ = .82, with significant differences between the iTracker and both of the other two, *t*s ≥ 11.07, *p*s < .001, *d*s ≥ 1.84, but no significant difference between the eTracker and MouseTracker, *t*(35) = 1.81, *p* = .079, *d =* 0.30. Compatibility effects differed between setups, *F*(2, 34) = 12.00, *p* < .001, η_p_^2^ = .41, with the iTracker producing significantly smaller differences (∆ = 12535 px^2^) compared to the eTracker (∆ = 33879 px^2^) and the MouseTracker (∆ = 27341 px^2^), again with significant differences between the iTracker and both of the other two, *t*s ≥ 3.86, *p*s < .001, *d*s ≥ 0.64, but no significant difference between the eTracker and MouseTracker, *t*(35) = 1.74, *p* = .091, *d =* 0.29. Sequential adaptation effects emerged, *F*(1, 35) = 32.95, *p* < .001, η_p_^2^ = .49. No other effects were significant, *F*s ≤ 3.17, *p*s ≥ .054.

#### Questionnaire

For the analysis of the questionnaire, the data of seven participants was excluded due to incompleteness or reports of false feedback during the experiments^8^. The handling of the iTracker (M = 7.48) was judged as overall easier than of the eTracker (M = 6.76), *t*(28) = 2.76, *p* = .010, *d* = 0.51, with the MouseTracker (M = 7.28) in between, *t*s ≤ 1.47, *p*s ≥ .154, *d*s ≤ 0.27. Ratings for overall pleasantness were led by the iTracker (M = 7.07), which differed significantly from both the MouseTracker (M = 6.14) and the eTracker (M = 5.72), *t*s ≥ 2.71, *p*s ≤ .011, *d*s ≥ 0.50, but with no differences between the latter setups, *t*(28) = 1.05, *p* = .304, *d* = 0.19. Similar results emerged from the judgment of movement fluidity, again led by the iTracker (M = 7.55), which again differed significantly from both the MouseTracker (M = 4.52) and the eTracker (M = 4.52), *t*s ≥ 4.98, *p*s < .001, *d*s ≥ 0.92, but no differences between the latter setups, |*t*| < 1. Movements on the iTracker were perceived as more effortless (M = 6.45) than on the eTracker (M = 4.93), *t*(28) = 2.22, *p* = .035, *d* = 0.41, with the MouseTracker (M = 5.28) in between, *t*s ≤ 1.66, *p*s ≥ .109, *d*s ≤ 0.31. However, the eTracker was found to be less prone to errors (M = 5.79) than the iTracker (M = 4.69), *t*(28) = 2.11, *p* = .043, *d* = 0.39, again with the MouseTracker (M = 5.72) in between, *t*s ≤ 1.77, *p*s ≥ .089, *d*s ≤ 0.32. There were no differences between the setups when it came to accessibility, *t*s ≤ 1.71, *p*s ≥ .099 *d*s ≤ 0.32, or perceived control of the movements, *t*s ≤ 1.03, *p*s ≥ .310 *d*s ≤ 0.19. More than half of the participants chose the iTracker (62.1%) if they had to do the experiment again, compared to the MouseTracker (20.7%) and the eTracker (17.2%).

### Discussion

In Experiment 1, we designed a Simon task and ran a version of the same experiment with three different setups. All setups produced main effects of compatibility, so in principle, all setups were able to measure the small temporal and spatial response differences between compatible and incompatible trials, which serves as a manipulation check.

Before deriving any concrete recommendations for how to design and analyze movement trajectory experiments in general (and not only for Simon tasks), our approach still begs the question of whether the results regarding the comparison of different setups would generalize to other experimental tasks. We believe that the Simon effect is a reasonable starting point from which to make informed recommendations, at least for experimental setups that aim at detecting spatial deviations towards one of two competing response options. Whether and how the suggestions that we describe here relate to other tasks still has to be explored in future research, but until such studies are available, we believe that the following can be used to arrive at informed design choices.

#### Measures, measures

As a first, coarse assessment, we discuss the overall data pattern to distill which measures best describe the effects of the experimental manipulation on participants’ movement trajectories, irrespective of the device these movements were performed on. Overall, we found large compatibility effects for all setups, specifically for SAs, MTs, and AUCs. As expected, the spatial response conflict in incompatible Simon trials slowed down responses and contorted movements towards the colored stimulus area (Hommel, 2011). The results for ITs are substantially weaker and suggest a moderate impact of compatibility at best (for the iTracker). The expected sequential adaptation effect, indicating reduced compatibility effects after an incompatible trial relative to after a compatible trial, also emerged for ITs, MTs, and AUCs, whereas for SAs this effect was (again) only found in the iTracker.^9^

Based on this summary, it is tempting to conclude that a measure (as well as an experimental setup) is more useful the more sensitive it is in detecting the almost “universal” Simon effect studied in this experiment. Yet, this assumption is probably premature, as other experimental effects may show a different profile. We thus do not intend to claim that, e.g., IT has no informative value, but quite the contrary. There might be settings in which this variable produces strong effects of interest (e.g., when placing less emphasis on speedy initiation). This again highlights that the choice of dependent variables is far from trivial for mouse- and finger-tracking experiments. Therefore, our ***Recommendation 1*** is that researchers should select their DVs according to the aim of the study (ideally before running the study) and justify their choice explicitly. A tailored set of DVs that is derived from the particular research question will be better suited to describe the cognitive processes that are to be studied, than applying a standard selection in each and every study.

#### Mouse- vs. finger-tracking

Even though all setups were principally able to detect the Simon effect, a closer look at the result pattern enables us to infer some unique properties of the different setups. The mouse-operated setups (MouseTracker and eTracker) show very quick response initiations (that were also unaffected by compatibility) compared to the finger-operated setup (iTracker), suggesting that with the former setups, participants start their responses with less planning than with the iTracker. This impression is further corroborated by the observation of initially “impulsive” movements (indicated by steeper SA; also earlier TTPV and TTPA) and overall less target-directed movement execution (indicated by higher MT and AUC; also higher MAD and Curv, more negative MinX, and more xFlips, see www.osf.io/am6yp) for mouse- versus finger-operated setups.

**Fig. 3:**
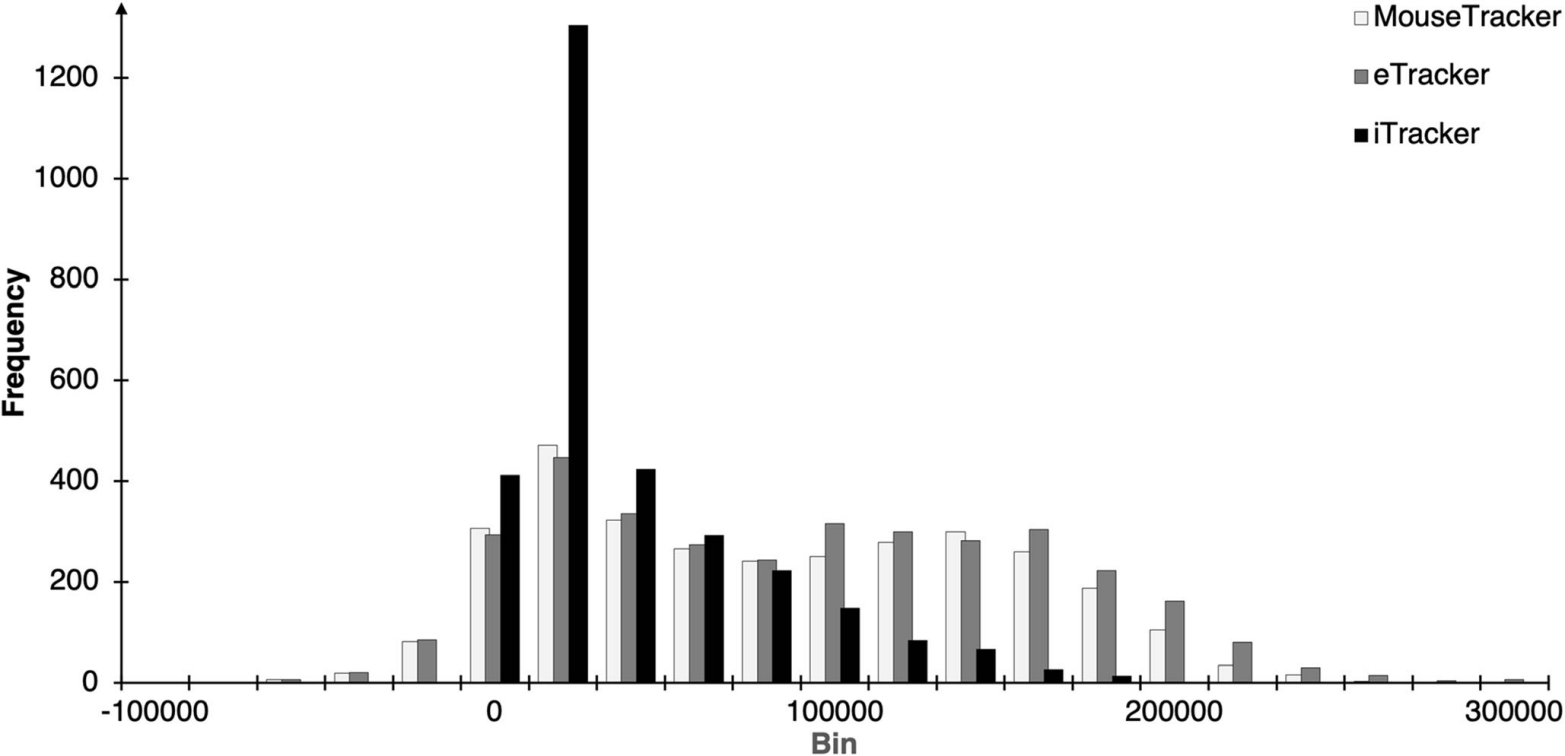
Histogram of the AUC distribution of incompatible trials in Experiment 1. On the *X*-axis, bin width is 20,000 px, and the label for each category represents the upper limit of the bin (e.g., the bin labeled “0” contains values of −19999 px^2^ to 0 px^2^). The *Y*-axis represents the number of cases in each bin, plotted separately for each setup

Furthermore, a lack of planning might have led to more biased movements to the wrong side in incompatible trials, combined with a change in direction towards the correct target area during response execution (indicated by higher compatibility effects for the mouse- versus the finger-operated setups in AUC; also in MinX, MAD, and Curv). In basic button press experiments, a considerable share of such insufficiently planned responses would most likely result in errors and would be removed from further analysis, but the tracking setup allows for correcting responses on the fly. In turn, this could explain the huge compatibility effects (in absolute terms) that are obtained with the MouseTracker and eTracker, as the trajectory for incompatible trials (and thereby the difference between incompatible and compatible trials) could be strongly driven by insufficiently planned and initially incorrect movements (for a similar argument, see Spivey, Grosjean, & Knoblich, 2005; Wulff, Haslbeck, Kieslich, Henninger, & Schulte-Mecklenbeck, 2019). Interestingly, larger compatibility effects emerged only when assessing the raw data of each device, in that both mouse-operated setups produced numerically larger AUCs and also a numerically larger absolute difference between the AUCs of the compatible and the incompatible conditions. When comparing the standardized effect sizes of the compatibility effect (see Fig. 2), all three setups were surprisingly comparable, suggesting that the larger deviations in the mouse-operated devices come at the expense of considerable additional variance, with one share of the trials producing very large deviations and the remaining trials producing only a small deflection. This argument is further supported by an exemplary inspection of the distribution of the AUC data of incompatible trials. The MouseTracker and eTracker clearly show a broader, almost bimodal distribution (see Fig. 3; kurtosis values of *k* ≤ −0.79, with negative kurtosis values suggesting bimodality, Darlington, 1970), indicating that two distinct cognitive processes make up this data, while the iTracker produces a unimodal ex-Gaussian distribution of AUC values (*k* = 1.77; for similar results, see Zhang, 2019).^10^

In contrast, the iTracker shows relatively slower response initiations (that are also affected by compatibility) and quicker response execution times, indicating that responses are more often properly selected prior to response initiation. This longer planning seems to lead to overall more target-directed movement trajectories that require less on-the-fly correction. Still, the iTracker clearly produces the compatibility effect that we tested for. The size of the compatibility effect is often smaller here (only in terms of absolute values, but not *d*_*z*_s, see Fig. 2); however, these differences are less driven by initiation errors. Speculatively, this difference might suggest a purer separation of planning and execution aspects of the responses with finger-movements as compared to the mouse-operated setups.

The question is now why a change of setups resulted in such qualitative differences in responding in almost identical tasks. Next to small design differences between the setups, such as the lack of feedback for slow responses with the MouseTracker, or the debatable choice of equating clicking the mouse with touching the screen and lifting the finger from the screen, a crucial difference between the touchscreen and the mouse setups is that the latter setups inherently involve a transformation of hand movements to cursor movements on the screen. We did our best to make this translation as direct as possible (with comparable speed and mouse acceleration turned off). However, what cannot be assimilated is that for the computer mouse, response location (on the table) and effect location (on the screen) are inherently different, whereas with the touchscreen, there is a prefect translation of finger movements to cursor changes, so that response and effect location are inherently identical. Furthermore, this perfect translation of finger and cursor changes also comes with a higher risk of omission error when the finger slips from the touchscreen, which cannot happen with the mouse.

Still, there remain differences that might have resulted in more liberal response execution with the computer mouse, even if that means that the cursor initially approached the wrong target area. However, if it was their own finger that moved on the screen, participants seemed to have been more cautious, taking care to avoid approaching the wrong target area or traveling long distances, increasing the risk of producing an omission error. This might have to do with a special sensitivity towards one’s own body (Costantini & Haggard, 2007) or even enhanced attention towards the area around one’s own hands (Reed, Grubb, & Steele, 2006; Taylor, Gozli, Chan, Huffman, & Pratt, 2015), but this qualitative difference between the setups deserves more research.

These observations allow for a second recommendation: ***Recommendation 2*** is that researchers aiming at a precise distinction of planning and execution processes are well-advised to use setups with direct finger-tracking such as the present touchscreen setup. By contrast, if the aim of an experiment is to show a difference between two conditions of interest—irrespective of whether this effect relates to errors in early action decisions and movement planning, or whether it relates to differences in how movements are executed—the mouse-operated setups may yield larger absolute differences between the conditions and possibly increased power.

#### Ready-made vs. custom-built

In addition to the pronounced differences between the two mouse-operated setups and the touchscreen setup, the two mouse-operated setups also differed systematically from each other, which might be driven by those design choices that could not be equalized between the two setups. For example, the MouseTracker does not allow for presenting custom feedback except for commission errors, i.e., there was no feedback for trials with slow response initiation or slow response execution. Another difference between the two mouse-operated setups was the shape of the areas, which are rectangular by design in the MouseTracker, whereas the eTracker used round areas. These small differences again show that even seemingly trivial design choices might influence how participants execute their movements to complete the task. This reasoning overall favors custom-built setups over general-purpose setups if programming skills allow for the former option to be used^11^.

#### Measurement precision

**Fig. 4:**
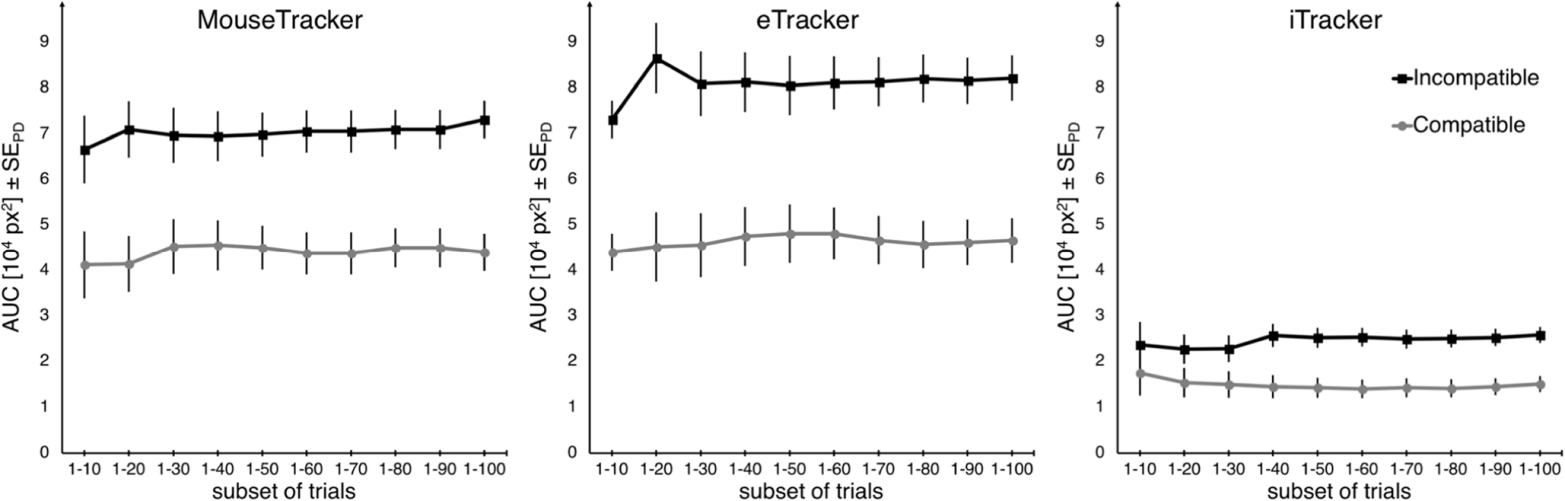
Illustration of the measurement precision. Mean AUCs (*Y*-axis), separate for each setup, and current compatibility (gray circles for compatible trials, black squares for incompatible trials) are plotted against each increasing subset of trials (*X*-axis)

Another question pertains to measurement precision for mouse- and finger-tracking setups (cf. Blinch, Kim, & Chua, 2018). That is: How many trials are required to properly measure the effect of interest? To approach this question empirically, we exemplarily analyzed the AUC data of each setup with an increasing subset of trials (Trial 1-10 vs. Trial 1-20 vs. Trial 1-30 and so on, up to Trial 1-100, which would constitute the complete first block of each setup; see Fig. 4). As the experiment did not include any practice trials, it is also interesting to see whether the smaller subsets differ from the larger ones, to see how fast participants become familiar with the setup.

Data shows that compatibility effects were significant for all subsets of trials, *t*s > 2.25, *p*s < .031, *d*s > 0.38, except for the smallest subset of the iTracker, *t*(34) = 1.19, *p* = .242, *d* = 0.20 (which is probably due to the high omission rate in this setup). The size of the compatibility effect was stable after 30 trials (which comes down to about 12 trials per design cell after errors and omissions are excluded), indicated by a lack of interaction between the factors compatibility and subset of trials when considering only subsets of 30 and larger for all devices, *F*s < 1.23, *p*s > .322. With this analysis, we do not want to advise researchers to run decreasingly short experiments with questionable trial numbers, but simply want to demonstrate that movement tracking can produce robust results even for cases in which measurements cannot be taken arbitrarily often or when the inclusion of a practice phase is difficult. Assuming effect sizes of those studied here, ***Recommendation 3*** is thus to opt for a minimum of about 10–15 analyzable trials per design cell whenever possible.

Note, however, that this is only an exploratory analysis, and that currently we use every participant’s first block per setup, irrespective of the order of setups that they worked on (e.g., their first, third, and fifth block, with the order of setups counterbalanced). A promising way to validate this analysis would be a between-subject comparison of the very first trials of an experiment, which our current experiment was not designed for. Still, we believe that this analysis is informative with regard to how fast each setup produces reliable measurements (rather than how fast they become familiar with the overall task requirements).

#### Subjective assessment

The questionnaire data further shows that handling of the iTracker was rated easiest, most pleasant, most fluid, and most effortless relative to the other setups. However, it was also judged as more prone to errors, which might stem from the fact that the iTracker introduced an additional type of error: response omissions via prematurely lifting the finger from the screen (for a means to address omission rates, see Experiment 4). Finally, the iTracker was also the setup that more than half of participants chose to work on if they had to do the experiment again. Taken together, the subjective assessment clearly favored the touchscreen device as an input method.

### Interim conclusions

The results of Experiment 1 suggest that, while all setups were able to detect the Simon effect, there are differences in how each setup captures the temporal and spatial dynamics of producing hand and finger movements. Touchscreen setups seem to be favored by the participants.

Based on these results, we decided to proceed with the iTracker version for the subsequent experiments. Another reason for this choice is that the iTracker is the newest of all devices and requires further validation. In each of the subsequent experiments, one of the design choices of the current iTracker setup was manipulated to see how it affects movement trajectories. Therefore, the experiments do not build upon each other, and can consequently be read in any order.

## Experiment 2: Spatial layout

### Introduction

In Experiment 2, we manipulated the spatial layout of the response locations. In Experiment 1, we chose a layout in which movements had to be directed rather upwards (target areas were located 600 px above and 300 px sideward of the starting area). This arguably leaves much freedom regarding execution, as, irrespective of the required response, movements can initially be directed upwards to postpone the decision process at only a small temporal and spatial cost (Wong & Haith, 2017). Other designs, however, employ wide layouts, with some even placing the target areas on the same vertical position as the starting areas (e.g., Buetti, Juan, Rinck, & Kerzel, 2012). With a wider layout, movements to the wrong target location are likely costlier, as the hand would have to travel longer distances to correct for initiation errors (Burk, Ingram, Franklin, Shadlen, & Wolpert, 2014). This might lead to a more cautious response strategy, and thus an expenditure of more time to decide where to move before moving, rather than to start moving early and decide on the eventual target location on the fly. In Experiment 2, we therefore tested how the factor layout influences movement trajectories.

### Methods

#### Participants

A set of twenty-four new participants were recruited (mean age = 28.2 years, *SD* = 10.5, 7 male, no left-handed) and were treated as in Experiment 1.

#### Apparatus, stimuli, and procedure

Experiment 2 was built on the iTracker version of Experiment 1. Stimuli, task, and instructions were unaltered, but the spatial layout of the experimental setup was manipulated: The *tall layout* had the target areas centered 400 px above and 200 px to the left and the right of the center of the starting area, so that the perfect line (see Fig. 1) produced an angle of 26.6° against the vertical midline. The *wide layout* placed the target areas 200 px above and 400 px left and right of the starting area, with the perfect line producing an angle of 63.4° against the vertical midline. That way, in both layouts, the distance between the center of the starting area and the centers of the target areas was identical, but the tall layout required a more upward-directed movement, while the wide layout required more sideward movements. To fit both layouts on the iPad screen, the experiment was run with the iPad in landscape mode (i.e., the longer side of the device is oriented horizontally). Participants worked on both layouts, layouts were manipulated between blocks, and the order of the layouts was counterbalanced between subjects.

A block consisted of 100 trials in randomized order, with an equal number of compatible and incompatible trials, and an equal number of required left and right responses. Participants completed four blocks overall, two blocks per layout, with short, self-paced breaks between blocks.

### Results

#### Data selection

Again we only omitted trials in which participants produced commission errors (5.8%) or omissions (15.1%). Errors were committed equally often in both conditions, *Χ*^*2*^(1) = 0.51, *p* = .475, but there were fewer omissions in the wide layout (10.9%) than in the tall layout (19.3%), *Χ*^*2*^(1) = 153.35, *p* < .001. The remaining data was left unfiltered, and preprocessing was conducted as in Experiment 1.

**Fig. 5:**
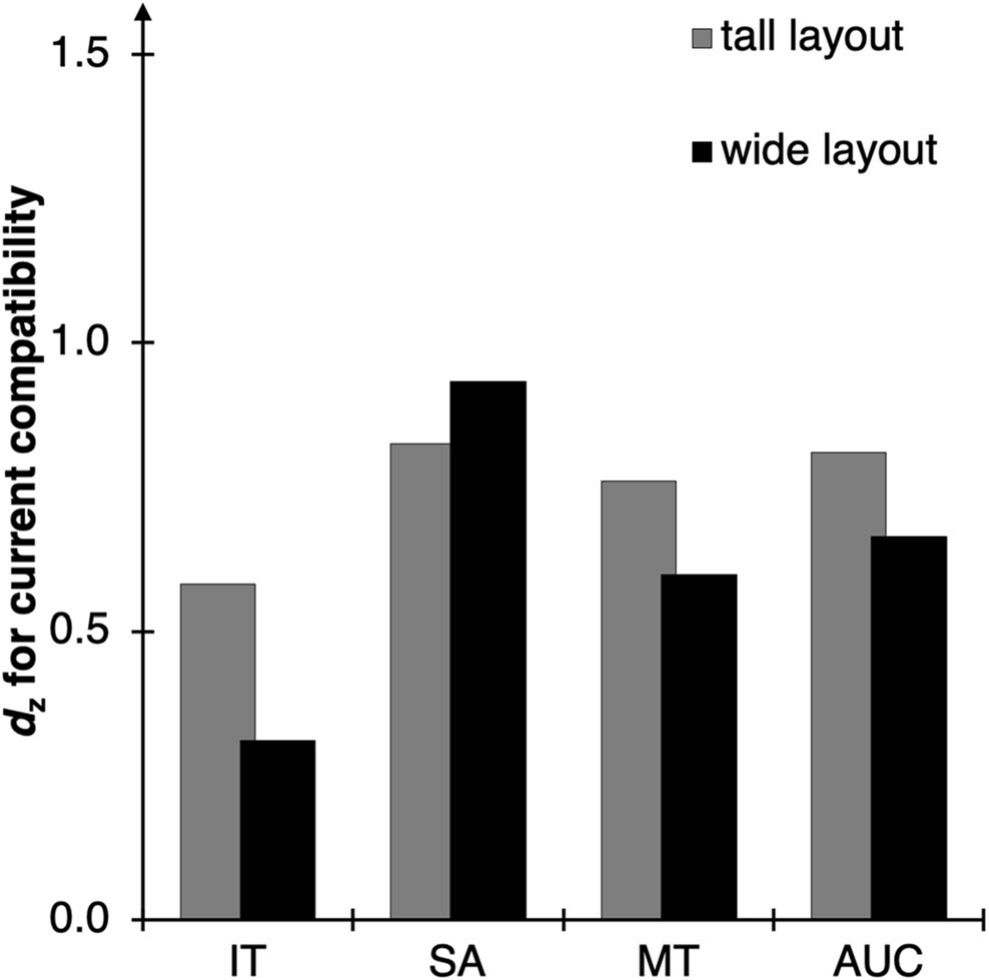
Main results of Experiment 2. Standardized effect sizes *d*_z_ for the effect of current compatibility (computed as current incompatible minus current compatible) separate for each DV (*X*-axis) and for each layout (columns). For the full results, see the Supplementary Material

ITs, SAs, MTs, and AUCs were then analyzed via 2 × 2 × 2 ANOVAs with current compatibility (trial N compatible vs. incompatible), preceding compatibility (trial N-1 compatible vs. incompatible), and layout (tall vs. wide) as within-subject factors (see Fig. 5). Again, we only scrutinized interactions with the factor layout in planned two-tailed *t* tests or separate ANOVAs. The full results with all DVs can be found in the Supplementary Material.

#### Initiation times

Data showed significantly faster response initiation for current compatible trials (460 ms) than for incompatible trials (475 ms), *F*(1, 23) = 10.74, *p* = .003, η_p_^2^ = .32. Sequential adaptation effects emerged, *F*(1, 23) = 22.51, *p* < .001, η_p_^2^ = .50. The factor layout produced neither a main effect nor any interaction, and no other effects were significant, *F*s ≤ 1.39, *p*s ≥ .250.

#### Starting angles

Data showed significantly steeper response initiation for current incompatible trials (18.9°) than for compatible trials (27.7°), *F*(1, 23) = 29.27, *p* < .001, η_p_^2^ = .56. Response initiation was steeper in the tall layout (12.3°) relative to the wide layout (34.2°), *F*(1, 23) = 111.29, *p* < .001, η_p_^2^ = .83. Compatibility effects differed between layouts, *F*(1, 23) = 5.36, *p* = .030, η_p_^2^ = .19, with the tall layout producing smaller differences (∆ = 5.6°) than the wide layout (∆ = 11.9°). Sequential adaptation effects emerged, *F*(1, 23) = 25.14, *p* < .001, η_p_^2^ = .52. No other effects were significant, *F*s ≤ 2.62, *p*s ≥ .119.

#### Movement times

Data showed significantly faster response execution for current compatible trials (311 ms) than for incompatible trials (331 ms), *F*(1, 23) = 17.78, *p* < .001, η_p_^2^ = .44. Sequential adaptation effects emerged, *F*(1, 23) = 60.34, *p* < .001, η_p_^2^ = .72. The factor layout produced neither a main effect nor any interaction, and no other effects were significant, *F*s ≤ 1.98, *p*s ≥ .173.

#### Area under the curve

Movements showed significantly greater spatial deviations for current incompatible trials (8519 px^2^) than for compatible trials (4823 px^2^), *F*(1, 23) = 17.12, *p* < .001, η_p_^2^ = .43, as well as after compatible trials (7283 px^2^) relative to after incompatible trials (6059 px^2^), *F*(1, 23) = 8.62, *p* = .007, η_p_^2^ = .27. Overall deviation was smaller in the tall layout (5245 px^2^) relative to the wide layout (8097 px^2^), *F*(1, 23) = 10.53, *p* = .004, η_p_^2^ = .31. Sequential adaptation effects emerged, *F*(1, 23) = 43.58, *p* < .001, η_p_^2^ = .66. No other effects were significant, *F*s ≤ 1.55, *p*s ≥ .226.

### Discussion

In Experiment 2, we manipulated the spatial layout of the movement-tracking setup, with a wide layout that required more sideward-directed movements, and a tall layout that required more upward-directed movements. First, what is surprising is the high omission rate with the tall layout. Closer inspection of the data revealed that this is mainly driven by some participants who started with the tall layout and had trouble understanding the instructions in the first trials. Movements were generally executed correctly overall, but the final landing position was just outside of the small target area in most of the trials. Therefore, we do not want to read too much into this difference (and address a possibility of reducing the omission rate in Experiment 4).

Overall, results show that there are no consistent qualitative differences for movement planning and movement execution between these two conditions other than the obvious (SA is steeper when the target areas are placed more upwards) and a smaller congruency effect in SA for the tall layout.

However, data suggests that the rare initiation errors are costlier in the wide layout than in the tall layout, indicated by the overall more extreme values for AUC (see also MinX and Curv in the Supplementary Material). As there is little room for uncertainty in the wide layout, the initial direction of movement had better be correct, as every inch traveled in the wrong direction must (almost) be traveled back in the case of initiation errors. The tall layout offers more freedom regarding movement execution, because participants can start their response upwards without necessarily making a decision right away. Still, responses do not seem to be initiated at a very low threshold (as probably in the mouse-operated setups of Experiment 1), so that the early phases of response planning can still be interpreted in a meaningful way and produce a significant compatibility effect. Hence, the increased degrees of freedom in executing movements in a tall relative to a wide layout encourages an online decision process, which lets us better observe the unfolding of the decision during movement execution (Scherbaum, Dshemuchadse, Fischer, & Goschke, 2010; for an attempt to delay the decision process even further, see Experiment 3). Somewhat surprisingly, the compatibility effect in IT was similar for the two layouts. Based on the idea that more processing occurs prior to movement start in the wide layout, we would have predicted even larger compatibility effects here.

Therefore, we do not want to give a strong recommendation when it comes to layout, as this design choice seems largely inconsequential, at least for the present experimental design. This is especially true when comparing the effect of the layout manipulation with the impact of different movement distances (see the following between-experiment analysis and Recommendation 4).

## Between-experiment analysis: Influence of movement extent

### Introduction

In the iTracker setup of Experiment 1, the target areas were 600 px above and 300 px left and right of the starting area. In the tall layout condition of Experiment 2, the target areas were 400 px above and 200 px left and right of the starting area. That way, the angle at which responses had to be initiated was identical, only the distance between the starting area and the target areas differed, with the *short distance* being two thirds of the *far distance*. Therefore, we decided to compare these two conditions to assess the impact of movement extent. Unlike all other design factors that are tested in this line of research, this is a between-participant comparison that extracts only one condition of each experiment rather than an active manipulation of the factor movement extent, and it neglects to take the order of conditions in each experiment into account. Therefore, this comparison is a post hoc test, though it may still provide informative suggestions for how to design a movement-tracking setup.

### Results

**Fig. 6:**
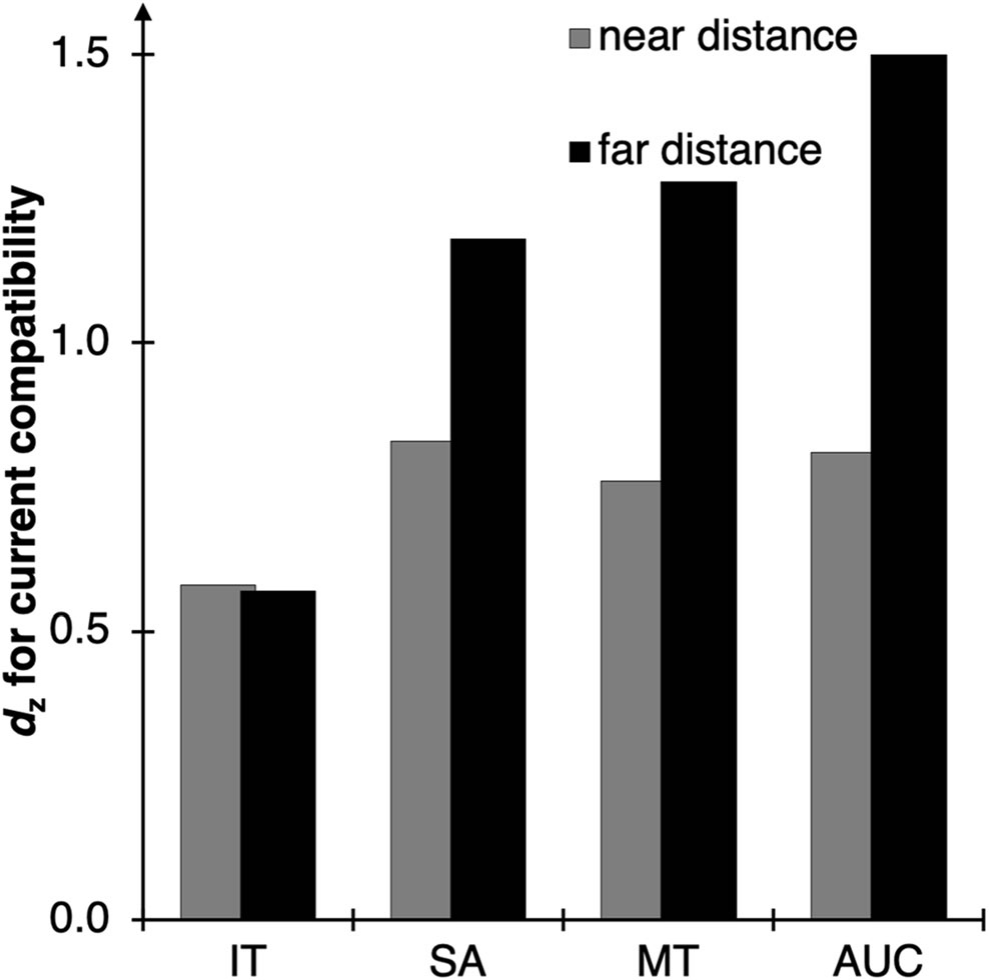
Results of the between-experiment analysis targeting movement extent. Standardized effect sizes *d*_z_ for the effect of current compatibility (computed as current incompatible minus current compatible) separate for each DV (*X*-axis) and for each distance (columns). For the full results, see the Supplementary Material

After removing all errors and omissions, ITs, SAs, MTs, and AUCs were analyzed via 2 × 2 × 2 ANOVAs with current compatibility (trial N compatible vs. incompatible) and preceding compatibility (trial N-1 compatible vs. incompatible) as within-subject factors, and distance (near vs. far) as a between-subject factor (see Fig. 6). We only scrutinized interactions with the factor distance in two-tailed *t* tests or separate ANOVAs. The full results with all DVs can be found in the Supplementary Material.

#### Initiation times

Data showed significantly faster response initiation for current compatible trials (406 ms) than for incompatible trials (422 ms), *F*(1, 58) = 20.38, *p* < .001, η_p_^2^ = .26. A main effect of distance, *F*(1, 58) = 9.31, *p* = .003, η_p_^2^ = .14, indicated faster response initiation with the far distance (380 ms) than with the near distance (465 ms). Sequential adaptation effects emerged, *F*(1, 58) = 20.20, *p* < .001, η_p_^2^ = .26. No other effects were significant, *F*s ≤ 1.37, *p*s ≥ .247.

#### Starting angles

Data showed significantly steeper response initiation for current incompatible trials (5.9°) than for compatible trials (12.2°), *F*(1, 58) = 57.01, *p* < .001, η_p_^2^ = .50, as well as after compatible trials (8.3°) compared to after incompatible trials (9.7°), *F*(1, 58) = 7.70, *p* = .007, η_p_^2^ = .12. Response initiation was steeper in the far distance (6.8°) relative to the near distance (12.3°), *F*(1, 58) = 10.96, *p* < .001, η_p_^2^ = .16. Sequential adaptation effects emerged, *F*(1, 58) = 27.78, *p* < .001, η_p_^2^ = .32. No other effects were significant, *F*s ≤ 1.70, *p*s ≥ .197.

#### Movement times

Data showed significantly faster response execution for current compatible trials (369 ms) than for incompatible trials (398 ms), *F*(1, 58) = 55.53, *p* < .001, η_p_^2^ = .49, as well as after compatible trials (380 ms) compared to after incompatible trials (387 ms), *F*(1, 58) = 4.19, *p* = .045, η_p_^2^ = .07. A main effect of distance, *F*(1, 58) = 12.53, *p* = .001, η_p_^2^ = .18, indicated faster response execution with the near distance (330 ms) than with the far distance (419 ms). Compatibility effects differed between distances, *F*(1, 58) = 8.07, *p* = .006, η_p_^2^ = .12, with the near distance producing significantly smaller differences (∆ = 17 ms) than the far distance (∆ = 38 ms). Sequential adaptation effects emerged, *F*(1, 58) = 58.39, *p* < .001, η_p_^2^ = .50. No other effects were significant, *F*s ≤ 1, *p*s ≥ .871.

#### Area under the curve

Movements showed significantly greater spatial deviations for current incompatible trials (20393 px^2^) than for compatible trials (11731 px^2^), *F*(1, 58) = 69.17, *p* < .001, η_p_^2^ = .54, as well as after compatible trials (17344 px^2^) relative to after incompatible trials (14779 px^2^), *F*(1, 58) = 14.37, *p* < .001, η_p_^2^ = .20. A main effect of distance, *F*(1, 58) = 49.99, *p* < .001, η_p_^2^ = .46, indicated larger spatial deviations with the far distance (23274 px^2^) than with the near distance (5245 px^2^). Compatibility effects differed between distances, *F*(1, 58) = 27.41, *p* < .001, η_p_^2^ = .45, with the near distance producing significantly smaller differences (∆ = 2850 px^2^) than the far distance (∆ = 12535 px^2^). Sequential adaptation effects emerged, *F*(1, 58) = 48.17, *p* < .001, η_p_^2^ = .45, and they were further modulated by distance, *F*(1, 58) = 8.79, *p* = .004, η_p_^2^ = .13, and sequential adaptation showed up for both distances, *F*s ≥ 30.24, *p*s < .001. Aftereffects did not differ between distances, *F*(1, 58) = 2.66, *p* = .108, η_p_^2^ = .04.

### Discussion

A comparison of the movement extent conditions (near vs. far) revealed that, next to the obvious influences (e.g., increasing MTs and AUCs with longer distances), unexpectedly, the overall time to complete a trial was almost identical between conditions (IT + MT + CT; near: 1210 ms, far: 1172 ms, |*t*| < 1). With the far distance, participants initiated their movements sooner, and spent more time on movement execution, which might have allowed the decision process to leave stronger traces on movement trajectories. With the near distance, there was less room for variation or on-the-fly corrections, which is why participants might have planned and selected their response properly in advance, resulting in smaller compatibility effects of the spatial parameters for the near distance. Therefore, as ***Recommendation 4***, we would advise the researcher to choose a longer distance, so that participants spend most of their time on movement execution rather than planning (whilst still producing meaningful results for the parameters mirroring the planning process), thereby allowing for maximum (natural) movement variation.

Compared to the influence of the factor layout (see Experiment 2), a larger movement extent seems to have a clear benefit over a smaller one, so that we would advise researchers to maximize the distance between starting area and target area with the hardware setup that is available rather than to insist on a specific geometric layout that might sacrifice valuable screen space.

## Experiment 3: Stimulus onset

### Introduction

Having addressed the factors setup, spatial layout, and movement extent, we will next focus on the time point of the stimulus onset. In the previous experiments, we displayed the stimulus color as soon as the starting area was touched, i.e., before movement initiation (also called *static* starting procedure). That gave participants time to plan their movement prior to its execution and determine some response parameters in advance.

Especially if one intended to use movement trajectories to study how attractions to certain response options evolve in time, one would ideally need to postpone the decision process to the movement execution station. This can be achieved by displaying the stimulus only after the movement has been initiated, or even only after a certain distance has been traveled (e.g., Scherbaum, Dshemuchadse, Fischer, & Goschke, 2010, also called a *dynamic* starting procedure), which forces participants to postpone the decision process to the movement execution stage^12^. As an upside, with this procedure we can be sure that the movement can capture every facet of the decision process. As a downside, this inherently means that the early stages of the movement, i.e., the data points prior to stimulus onset, cannot be analyzed in a meaningful way. In Experiment 3, we tested how the onset procedure affects participants’ movement trajectories. This experiment is similar to the work of Scherbaum and Kieslich (2018), which also varied the onset condition between groups of participants, but here we used a within-subject manipulation, and we used three rather than two different onset conditions.

### Methods

#### Participants

A set of twenty-four new participants were recruited (mean age = 24.3 years, *SD* = 2.7, 3 male, 2 left-handed) and were treated as in the previous experiments. The data for one participant was removed from the sample, as this participant reported having difficulty in handling the iPad after testing.

#### Apparatus, stimuli, and procedure

Experiment 3 was built on the iTracker version of Experiment 1. This time, we varied the time point of stimulus onset. As before, the target areas (and with them, the stimulus color) could be displayed before movement initiation (*before* condition: when the starting area was touched), but now we also designed conditions in which the target areas and stimulus color appeared simultaneously with movement initiation (*simultaneous* condition: when leaving the starting area), or after having traveled about 20% (120 px) of the distance (*subsequent* condition). Participants worked on all three stimulus onset conditions, which were manipulated between blocks, and the order of the stimulus onset conditions was counterbalanced between subjects.

A block consisted of 100 trials in randomized order, with an equal number of compatible and incompatible trials, and an equal number of required left and right responses. Participants completed six blocks overall, two blocks per stimulus onset condition, with short, self-paced breaks between blocks.

### Results

#### Data selection

Again we only omitted trials in which participants produced commission errors (3.9%) or omissions (11.4%). Fewer errors were committed in the subsequent condition (3.2%) than in the before (4.2%) or simultaneous condition (4.3%), *Χ*^*2*^(1) ≥ 5.48, *p*s ≤ .020, whereas the latter two did not differ from each other, *Χ*^*2*^(1) = 0.17, *p* = .676. There were no differences in omission rates, *Χ*^*2*^(1) ≤ 0.64, *p*s ≥ .426. The remaining data was left unfiltered, and preprocessing was conducted as in the previous experiments.

**Fig. 7:**
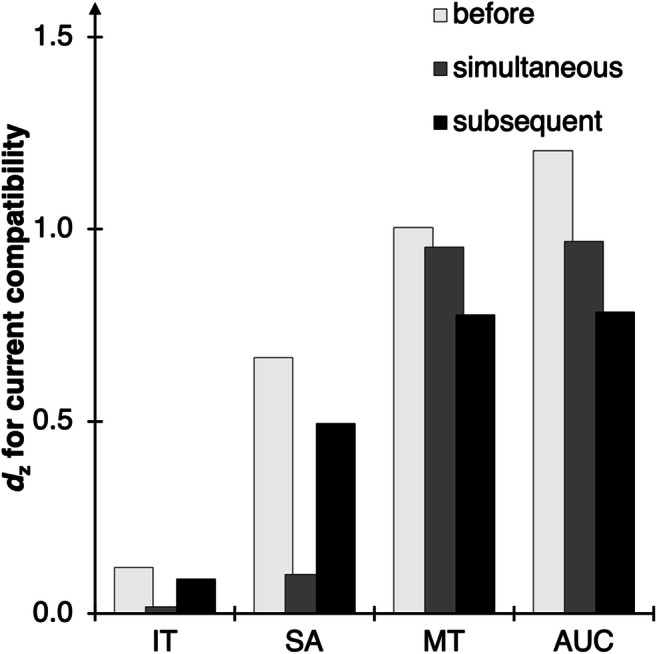
Main results of Experiment 3. Standardized effect sizes *d*_z_ for the effect of current compatibility (computed as current incompatible minus current compatible) separate for each DV (*X*-axis) and for each stimulus onset condition (columns). For the full results, see the Supplementary Material

ITs, SAs, MTs, and AUCs were then analyzed via 2 × 2 × 3 ANOVAs with current compatibility (trial N compatible vs. incompatible), preceding compatibility (trial N-1 compatible vs. incompatible), and stimulus onset (before vs. simultaneous to vs. subsequent to movement initiation) as within-subject factors (see Fig. 7). We only scrutinized interactions with the factor stimulus onset in planned two-tailed *t* tests or separate ANOVAs. The full results with all DVs can be found in the Supplementary Material.

#### Initiation times

Response initiation was slower with stimulus onset before movement initiation (253 ms) relative to the simultaneous (134 ms) and subsequent conditions (118 ms), *F*(2, 21) = 9.16, *p* = .001, η_p_^2^ = .47, with significant differences between the before condition and the others, *t*s ≥ 3.55, *p*s ≤ .001, *d*s ≥ 0.59, but no significant difference between the simultaneous and subsequent conditions, *t*(22) = 1.25, *p* = .219, *d* = 0.21. Sequential adaptation effects emerged, *F*(1, 22) = 4.46, *p* = .046, η_p_^2^ = .17. No other effects were significant, *F*s ≤ 1.08, *p*s ≥ .359.

#### Starting angles

Data showed significantly steeper response initiation for current incompatible trials (2.4°) than for compatible trials (4.4°), *F*(1, 22) = 13.60, *p* = .001, η_p_^2^ = .38. Compatibility effects differed between conditions, *F*(2, 21) = 3.58, *p* = .046, η_p_^2^ = .25, with the before condition producing significantly larger results (∆ = 3.8°) compared to the simultaneous condition (∆ = 0.2°), *t*(22) = 2.54, *p* = .016, *d* = 0.42, and the subsequent condition (∆ = 1.7°) in between, *t*s ≤ 1.72, *p*s ≥ .094, *d*s ≤ 0.29. Sequential adaptation effects emerged, *F*(1, 22) = 11.20, *p* = .003, η_p_^2^ = .12. No other effects were significant, *F*s ≤ 1.71, *p*s ≥ .206.

#### Movement times

Data showed significantly faster response execution for current compatible trials (575 ms) than for incompatible trials (610 ms), *F*(1, 22) = 33.22, *p* < .001, η_p_^2^ = .60. Response execution was faster with stimulus onset before movement initiation (486 ms) relative to the simultaneous (621 ms) and subsequent conditions (671 ms), *F*(2, 21) = 11.90, *p* < .001, η_p_^2^ = .53, with significant differences between the before condition and the others, *t*s ≥ 4.05, *p*s < .001, *d*s ≥ 0.68, but no significant difference between the simultaneous and subsequent conditions, *t*(22) = 1.54, *p* = .132, *d* = 0.26. Sequential adaptation effects emerged, *F*(1, 22) = 49.70, *p* < .001, η_p_^2^ = .69. No other effects were significant, *F*s ≤ 2.76, *p*s ≥ .086.

#### Area under the curve

Movements showed significantly greater spatial deviations for current incompatible trials (43197 px^2^) than for compatible trials (36139 px^2^), *F*(1, 22) = 32.80, *p* < .001, η_p_^2^ = .60, as well as after compatible trials (40282 px^2^) relative to after incompatible trials (39054 px^2^), *F*(1, 22) = 5.47, *p* = .029, η_p_^2^ = .20. Compatibility effects differed between conditions, *F*(2, 21) = 7.30, *p* = .004, η_p_^2^ = .41, with the before condition producing significantly larger results (∆ = 10006 px^2^) than with stimulus onset simultaneous to (∆ = 5840 px^2^) or subsequent to movement initiation (∆ = 5329 px^2^), *t*s ≥ 2.84, *p*s ≤ .008, *d*s ≥ 0.47, but no significant difference between the simultaneous and subsequent conditions, |*t*| < 1. Sequential adaptation effects emerged, *F*(1, 22) = 31.19, *p* < .001, η_p_^2^ = .59, and they were further modulated by stimulus onset, *F*(2, 21) = 4.36, *p* = .026, η_p_^2^ = .29, and sequential adaptation showed up for all conditions, *F*s ≥ 9.29, *p*s ≤ .006. No other effects were significant, *F*s ≤ 1.70, *p*s ≥ .207.

### Discussion

In Experiment 3, we tested how the stimulus onset prior to, simultaneous to, or subsequent to movement initiation affects movement trajectories. Data shows that with the simultaneous and subsequent conditions, participants start their movements more impulsively than with the before condition, as there is no stimulus to base their response on. Therefore, the early stages of the movement cannot be interpreted in a meaningful way. In contrast, SAs produce a significant compatibility effect only in the before condition, and AUC effects are also strongest in the before conditions, which might be driven by a systematic pull towards the alternative response option in the case of incompatible trials during the course of the whole movement rather than only towards the end. For the temporal markers, the duration of the whole response does not differ between the conditions (IT + MT + CT; before: 1025 ms, simultaneous: 1226 ms, subsequent: 1304 ms; |*t*|s < 1.67, *p*s > .105, *d*s < 0.34), so that there is no overall benefit from having participants initiate their movements quickly. Matters might change when using stricter deadlines, however. In the present setup, participants were to initiate their movement within 1000 ms and execute it within 1500 ms. Individual response initiation and execution times were far below these limits in most of the trials; shortening such deadlines likely would result in a more similar pattern of results across the three conditions.

Furthermore, overall spatial distortions as captured via mean AUC were similar across conditions. This suggests that the data of the before condition comprises a larger share of effects of interest, with more curved trajectories in the incompatible condition and more direct trajectories in the compatible condition, whereas the measurements of both of the other conditions comprise more unsystematic noise, as participants have to opt for intermediate movements at first. Because of such intermediate movements in the early phases of the trajectory, relative differences between the compatible and the incompatible condition are also necessarily inflated by differences in decision time after target onset, so that effects on AUC likely comprise both spatial and temporal factors when presenting the target on the fly.

Based on these considerations, ***Recommendation 5*** is the following: If one intends to study spatial parameters of movement trajectories such as AUCs, MAD, or Curv, the target should ideally be presented before movement onset. Presenting the target on the fly, by contrast, might be more viable if one intends to study the dynamics of the movement trajectory such as X flips or entropy, even though this is purely speculative at this point, as even the DVs that capture movement dynamics did not favor the dynamic starting procedures.

## Experiment 4: Target size

### Introduction

Next, we will have a look at the factor target size. The previous experiments produced rather high omission rates, which might indicate that the target areas are quite difficult to hit, being rather small. According to Fitts’ law (Fitts, 1954), increasing the target size should speed up movements, because less precision is required. However, with larger target areas, movements could become more scattered, as there is a larger area in which they can end, which might decrease the statistical power due to a higher variance. In Experiment 4, we therefore tested how the size of the target areas influences performance.

### Methods

#### Participants

A set of twenty-four new participants were recruited (mean age = 27.3 years, *SD* = 10.0, 7 male, 3 left-handed) and were treated as in the previous experiments.

#### Apparatus, stimuli, and procedure

Experiment 4 again was built on the iTracker version of Experiment 1. This time, we varied the size of the target area. As before, the target areas could be 60 px in diameter (*small target size*), and now we added target areas of 120 px (*medium target size*) and 180 px (*large target size*) in diameter (smaller areas were not tested due to the considerable number of omissions with target areas of 60 px in diameter in the previous experiments). The center of the target areas stayed the same in all size conditions. Participants worked on all three size conditions, which were manipulated between blocks, and the order of the size conditions was counterbalanced between subjects.

A block consisted of 100 trials in randomized order, with an equal number of compatible and incompatible trials, and an equal number of required left and right responses. Participants completed six blocks overall, two blocks per size condition, with short, self-paced breaks between blocks.

### Results

#### Data selection

Again we only omitted trials in which participants produced commission errors (5.6%) or omissions (6.3%). More errors were committed in the large target condition (6.8%) than in the small (4.9%) or medium condition (5.1%), *Χ*^*2*^(1) ≥ 16.07, *p*s < .001, whereas the latter two did not differ from each other, *Χ*^*2*^(1) = 0.17, *p* = .676. Omissions were committed more often in the small target condition (11.0%) than in the large (3.8%) or medium condition (4.2%), *Χ*^*2*^(1) ≥ 172.18, *p*s < .001, whereas the latter two did not differ from each other, *Χ*^*2*^(1) = 1.43, *p* = .232. The remaining data was left unfiltered, and preprocessing was conducted as in the previous experiments.

**Fig. 8:**
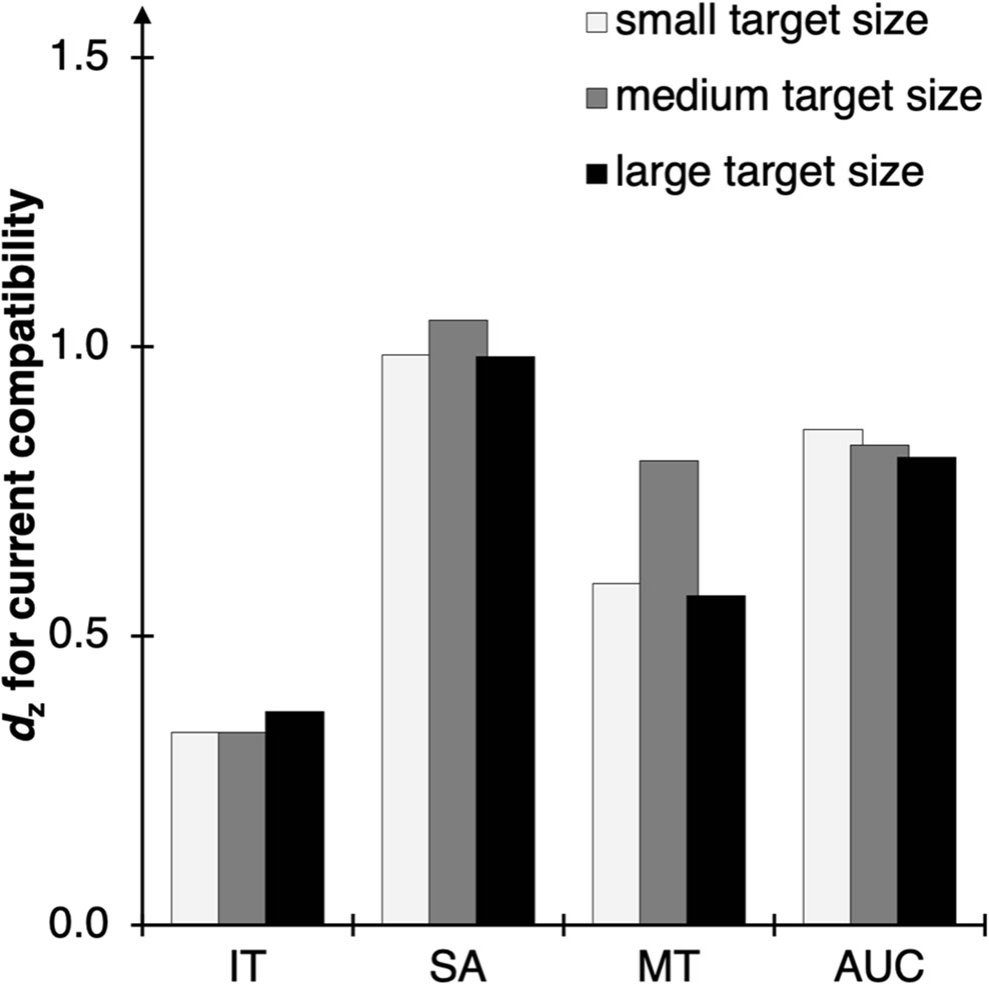
Main results of Experiment 4. Standardized effect sizes *d*_z_ for the effect of current compatibility (computed as current incompatible minus current compatible) separate for each DV (*X*-axis) and for each target size (columns). For the full results, see the Supplementary Material

ITs, SAs, MTs, and AUCs were then analyzed via 2 × 2 × 3 ANOVAs with current compatibility (trial N compatible vs. incompatible), preceding compatibility (trial N-1 compatible vs. incompatible), and target size (small vs. medium vs. large) as within-subject factors (see Fig. 8). We only scrutinized interactions with the factor target size in planned two-tailed *t* tests or separate ANOVAs. The full results with all DVs can be found in the Supplementary Material.

#### Initiation times

Response initiation was faster after compatible trials (396 ms) than after incompatible trials (407 ms), *F*(1, 23) = 14.47, *p* = .012, η_p_^2^ = .25. Sequential adaptation effects emerged, *F*(1, 23) = 17.43, *p* < .001, η_p_^2^ = .43, and they were further modulated by target size, *F*(2, 22) = 5.32, *p* = .013, η_p_^2^ = .33, and sequential adaptation showed up only for the small and large targets, *F*s ≥ 8.37, *p*s < .008, and not with medium-sized targets, *F*(1, 23) = 1.60, *p* = .219, η_p_^2^ = .07. No other effects were significant, *F*s ≤ 3.38, *p*s ≥ .079.

#### Starting angles

Data showed significantly steeper response initiation for current incompatible trials (6.9°) than for compatible trials (12.0°), *F*(1, 23) = 42.29, *p* < .001, η_p_^2^ = .65, as well as after compatible trials (9.9°) relative to after incompatible trials (9.0°), *F*(1, 23) = 5.00, *p* = .035, η_p_^2^ = .18. Sequential adaptation effects emerged, *F*(1, 23) = 35.88, *p* < .001, η_p_^2^ = .61. No other effects were significant, *F*s ≤ 2.29, *p*s ≥ .125.

#### Movement times

Data showed significantly faster response execution for current compatible trials (339 ms) than for incompatible trials (359 ms), *F*(1, 23) = 19.05, *p* < .001, η_p_^2^ = .45. Response execution was slower with small targets (412 ms) relative to medium (332 ms) and large targets (303 ms), *F*(2, 22) = 38.26, *p* < .001, η_p_^2^ = .78, with significant differences between all conditions, *t*s ≥ 3.12, *p*s < .001, *d*s ≥ 0.52. Sequential adaptation effects emerged, *F*(1, 23) = 49.04, *p* < .001, η_p_^2^ = .68. No other effects were significant, *F*s ≤ 1.18, *p*s ≥ .289.

#### Area under the curve

Movements showed significantly greater spatial deviations for current incompatible trials (21042 px^2^) than for compatible trials (15089 px^2^), *F*(1, 23) = 28.87, *p* < .001, η_p_^2^ = .56. Sequential adaptation effects emerged, *F*(1, 23) = 51.81, *p* < .001, η_p_^2^ = .69. No other effects were significant, *F*s ≤ 1.98, *p*s ≥ .173.

### Discussion

In Experiment 4, we varied the target size in our setup. At a first glance, this manipulation does not seem to affect movement trajectories in a fundamental way other than the expected influence on movement times and spatial precision (Fitts, 1954), with faster and more accurate movements for larger target areas. Interestingly, even with the large target areas, participants seemed to aim at the center rather than lifting their finger as soon as some point within the target area had been reached (see also the results for FDT in the Supplementary Material, even with the large target areas, participants landed on average within the perimeter of the small target area)^13^. Based on our results, as ***Recommendation 6***, we would advise the use of medium-sized target areas (with an index of difficulty of ID=1.05 here; Fitts, 1954), as they represent a good compromise when trading off omission rate for spatial precision requirements. As an alternative, one might also display small target areas during the experiment (e.g., 60 px in diameter), but when processing the data, use virtual target areas that are somewhat larger (e.g., 100 px in diameter). That way, all movements that fell just short of being accurate enough for the small targets can still be included in the analysis, but because the small target areas are displayed, the experiment still requires high spatial precision, increasing uniformity at the beginning and end of the trajectories, so that variations during the proper execution can be best observed.

## Experiment 5: Hit detection

### Introduction

In the final experiment of this series, we will have a look at how target hits are detected. In all our previous experiments, hits were detected when the target area was clicked or the finger was lifted from the screen, within the bounds of the target area, which requires high spatial precision at the end of the movement (especially with small target areas, see Experiment 4). In contrast to this so-called *click* condition (terminology taken from the MouseTracker, although the touchscreen devices require *lifting* the finger from the screen, e.g., Foerster, Wirth, Herbort, Kunde, & Pfister, 2017; Wirth, Foerster, Herbort, Kunde, & Pfister, 2018), some setups simply require participants to hit one of the target areas during their movement, but the movement does not necessarily have to end within the target area (*hover* condition in the MouseTracker, e.g., Scherbaum, Dshemuchadse, Fischer, & Goschke, 2010). That way, movements do not have to be slowed down at the end to produce a successful response, which might provide more direct access to the movement dynamics of the decision process. However, allowing participants to end their movement anywhere (as long as one of the target areas was hit at some point during the movement) could produce more variable movement trajectories, which might decrease statistical power. In this experiment, we therefore compared the two methods for detecting target hits.

### Methods

#### Participants

A set of twenty-four new participants were recruited (mean age = 29.1 years, *SD* = 8.9, 8 male, 3 left-handed) and were treated as in the previous experiments.

#### Apparatus, stimuli, and procedure

Experiment 5 again was built on the iTracker version of Experiment 1. This time, we varied the conditions for a movement to count as a target hit. As before, one condition could require that a movement is finished by lifting the finger from the screen within one of the target areas (*lift* condition), whereas another condition would just require that the correct target area is touched during the movement, but the finger could also be lifted outside the target area (*touch* condition). In both cases, a trial would be completed with the release of the finger, but target hits would be detected based on different criteria. In this way, the affordance on the spatial precision is varied, with the lift condition requiring more precise movements. Participants worked on both hit conditions, which were manipulated between blocks, and the order of the hit conditions was counterbalanced between subjects. As with the manipulation of the hit condition, both lift and touch conditions looked identical, we placed small written markers (lift vs. touch) in the lower right corner of the screen so participants would know how hits were detected in each block.

A block consisted of 100 trials in randomized order, with an equal number of compatible and incompatible trials, and an equal number of required left and right responses. Participants completed four blocks overall, two blocks per hit condition, with short, self-paced breaks between blocks.

### Results

#### Data selection

Again we only omitted trials in which participants produced commission errors (3.4%) or omissions (7.1%). Errors were more prominent in the lift condition (4.3%) than in the touch condition (2.5%), *Χ*^*2*^(1) = 26.24, *p* < .001, and there were more omissions in the lift condition (8.5%) than in the touch condition (5.6%), *Χ*^*2*^(1) = 30.13, *p* < .001. The remaining data was left unfiltered, and preprocessing was conducted as in the previous experiments.

**Fig. 9:**
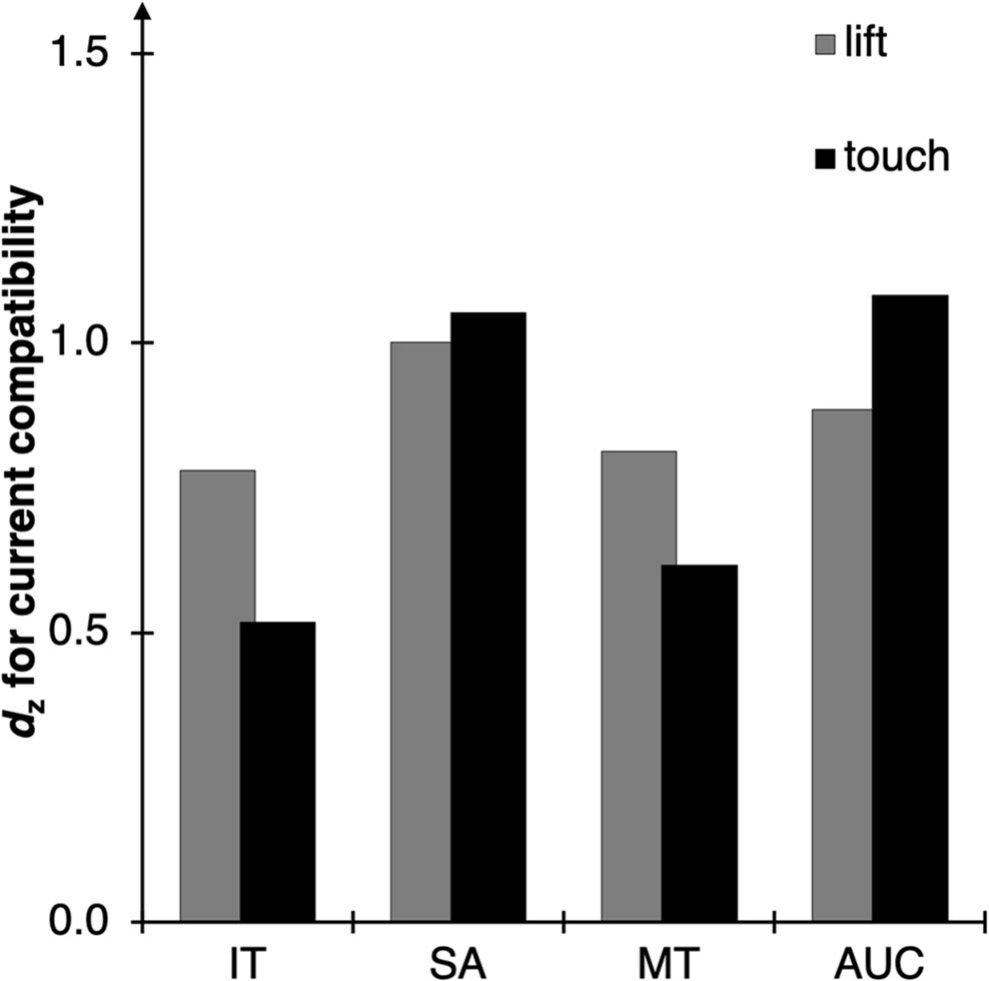
Main results of Experiment 5. Standardized effect sizes *d*_z_ for the effect of current compatibility (computed as current incompatible minus current compatible) separate for each DV (*X*-axis) and for each hit condition (columns). For the full results, see the Supplementary Material

ITs, SAs, MTs, and AUCs were then analyzed via 2 × 2 × 2 ANOVAs with current compatibility (trial N compatible vs. incompatible), preceding compatibility (trial N-1 compatible vs. incompatible), and hit condition (lift vs. touch) as within-subject factors (see Fig. 9). Again, we only scrutinized interactions with the factor hit condition in planned two-tailed *t* tests or separate ANOVAs. The full results with all DVs can be found in the Supplementary Material.

#### Initiation times

Data showed significantly faster response initiation for current compatible trials (377 ms) than for incompatible trials (390 ms), *F*(1, 23) = 20.75, *p* < .001, η_p_^2^ = .47, as well as after compatible trials (378 ms) relative to after incompatible trials (389 ms), *F*(1, 23) = 18.63, *p* < .001, η_p_^2^ = .45. Sequential adaptation effects emerged, *F*(1, 23) = 10.54, *p* = .004, η_p_^2^ = .31. The factor hit condition produced neither a main effect nor any interaction, and no other effects were significant, *F*s < 1.

#### Starting angles

Data showed significantly steeper response initiation for current incompatible trials (3.7°) than for compatible trials (9.1°), *F*(1, 23) = 41.60, *p* < .001, η_p_^2^ = .64. Sequential adaptation effects emerged, *F*(1, 23) = 19.66, *p* < .001, η_p_^2^ = .46. No other effects were significant, *F*s ≤ 1.94, *p*s ≥ .177.

#### Movement times

Data showed significantly faster response execution for current compatible trials (413 ms) than for incompatible trials (438 ms), *F*(1, 23) = 21.70, *p* < .001, η_p_^2^ = .49, as well as after compatible trials (430 ms) relative to after incompatible trials (420 ms), *F*(1, 23) = 6.72, *p* = .016, η_p_^2^ = .23. Response execution was overall faster in the touch condition (388 ms) than in the lift condition (462 ms), *F*(1, 23) = 17.39, *p* < .001, η_p_^2^ = .43. Sequential adaptation effects emerged, *F*(1, 23) = 55.24, *p* < .001, η_p_^2^ = .71. No other effects were significant, *F*s < 1.

#### Area under the curve

Movements showed significantly greater spatial deviations for current incompatible trials (26035 px^2^) than for compatible trials (18957 px^2^), *F*(1, 23) = 34.24, *p* < .001, η_p_^2^ = .60, as well as after compatible trials (23782 px^2^) relative to after incompatible trials (21210 px^2^), *F*(1, 23) = 16.20, *p* = .001, η_p_^2^ = .41. Sequential adaptation effects emerged, *F*(1, 23) = 42.26, *p* < .001, η_p_^2^ = .64. No other effects were significant, *F*s ≤ 1.04, *p*s ≥ .318.

### Discussion

In Experiment 5, we tested different methods of how target hits were detected. In the lift condition, participants had to end their movement within a target area, whereas in the touch condition, they simply had to hit one of the target areas during their movement without necessarily ending their movement within its confines. In the touch condition, participants were quicker to execute their movement and release their finger from the screen after hitting a target (see CTs in the Supplementary Material). And obviously, when participants are no longer required to end their movements within the target area, the residual distance to the target center is larger in the touch condition (see FDTs in the Supplementary Material). Other than that, hit condition does not seem to influence participants’ movement behavior in a fundamental way. However, the touch condition produces fewer omissions, due to the lower spatial precision that is required to successfully complete a trial. So as ***Recommendation 7***, we suggest that the touch condition can be a good alternative to larger target areas (see Experiment 4) to reduce omission rates.

Contrarily, a recent study found less consistent movement trajectories with the hover condition than with the click condition (Schoemann, Lüken, Grage, Kieslich, & Scherbaum, 2019). Therefore, keep in mind that this recommendation might be very specifically tailored to the current task and to finger-tracking on touchscreen devices.

We allowed participants to continue their movement after a target had been hit in the touch condition to be able to analyze the final stages of the movement and derive DVs such as FDT. However, that way, participants did not receive clear feedback about whether they ultimately hit a target during their movement. So, in the touch condition (or the hover condition in the MouseTracker), one might also terminate a trial as soon as a target is hit, to clearly signal a successful response. Of course, this is only possible if markers such as the residual distance to the target are not essential to the current research question. Also, it is important to consider how and when measures are taken. Our approach here is to measure MT as soon as the perimeter of the target area is crossed, irrespective of the hit condition. Typically, the hover condition uses the same criteria, neglecting anything that happens after entering the target area. But in the click condition, MT is usually only logged when the target area is clicked, not differentiating between MT and CT. This difference should be kept in mind not only when designing movement-tracking experiments, but also when interpreting the corresponding data.

## Conclusion

The current line of research was conducted with two goals in mind: first, to test how design choices can influence movement trajectories in typical mouse- and finger-tracking tasks, and second, to give an overview and evaluation of the most widely used dependent measures that can be derived from the corresponding movement trajectories. Overall, the setup that we used produced robust Simon effects (as expected), which is a crucial manipulation check. Based on how the Simon effect varied across devices and based on how it was affected by different design choices, our observations yield several recommendations for designing and analyzing mouse- and finger-tracking experiments:

- **Measures:** First, choose a set of dependent measures that reflect your research question and the cognitive processes that you assume to be at work. The choice of relevant measures may even dictate some of the subsequent design choices.
- **Input device:** For showing any effect between two conditions, mouse-operated setups might yield higher effect sizes than finger-tracking setups, which renders mouse-operated setups ideal for proof-of-principle investigations. If a researcher aims for more stringent distinction between planning and execution processes, touchscreen devices should be the input method of choice.
- **Number of trials:** Design your experiment so that each design cell consists of at least 10–15 trials, after errors and outliers have been removed. Errors and outliers can be reduced by employing a practice block, providing useful feedback, and adjusting the task affordance via the other design parameters discussed here.
- **Target distance:** Place the target areas as far from the starting area as the setup allows. With longer movements, minute spatial differences can be observed more easily. Choose a display layout (tall vs. wide) that allows for maximized movement distances.
- **Stimulus onset:** If dynamic changes of the mind are of central interest, displaying the stimulus only after a movement has been initiated can postpone the decision process to the movement execution stage. If the overall temporal and spatial parameters of the movement trajectory are of interest, displaying the stimulus before movement initiation additionally allows for a meaningful analysis of the planning and early movement phases.
- **Target size:** Choose a target size that does not require overly high spatial precision, to prevent response omissions. Especially with touchscreen devices, keep in mind that the finger obscures the response location, so that the diameter of the target area should ideally be larger than the fingertip.
- **Hit detection:** As an alternative to adjusting target size to reduce response omissions, you can also detect target hits based on a touch instead of a lift criterion. Keep in mind that with the variation of these detection methods, the theoretical constructs captured by your measures might differ as well.

When it comes to dependent measures, we consistently observed robust effects for spatial parameters of the movement as represented by summary measures such as AUC and also in early markers such as SAs. Other measures such as ITs or measures relating to velocity and acceleration (see Supplementary Material online) were less affected. This pattern mirrors the strong focus of the Simon effect on spatial response conflict (Hommel, 2011). As highlighted in the discussion of Experiment 1, this pattern should not be taken to suggest that variables such as AUCs and SAs are generally to be preferred over other measures. Instead, the choice of parameters should ideally reflect the aim of the research at hand. If, for example, dynamic changes of the mind are of central interest, one would ideally opt for measures such as X flips or overall movement entropy. These measures account for directional changes during movement execution and ideally capture evidence of decision uncertainty and potential changes of mind more clearly than AUCs and SAs. But if the overall movement behavior of participants is of interest, (a set of) measures that reflect the temporal and spatial characteristics of a trajectory should be chosen. To give a short but comprehensive overview, we decided on the measures IT, MT, SA, and AUC to report in the main text, as we believe they capture both the temporal and spatial properties of both the early and the later movement phases. Still, different research questions might require different measures, which is why we decided to analyze the whole range of measures for all experiments and provide these analyses online. This should enable informed decisions regarding which particular measures to compute when planning a conceptually similar experiment, and it allows for gauging how these measures are affected by design choices. Further, we also provide the raw data for more specialized approaches (e.g., Joch et al., 2019; Scherbaum et al., 2010), as well as the analysis scripts, so every step of the analysis can be easily reproduced.

For the design choices when planning a movement-tracking experiment, note that all recommendations are based on a particular task (the Simon effect with two response options), and all except the first one were tested specifically for a finger-tracking setup. Whether our conclusions generalize to other tasks and other input devices should be evaluated in future research (e.g., Scherbaum & Kieslich, 2018; Kieslich, Schoemann, Grage, Hepp, & Scherbaum, 2020). Until such studies are available, we believe that the current set of recommendations can be used to arrive at informed design choices that might make or break the detection of any given effect, so each parameter should be selected carefully. With our recommendations above, we do not wish to instate a “gold standard” for designing any type of movement-tracking experiment, but we acknowledge that different research methods are suitable for different research questions. Therefore, it is also crucial to be aware of how these design choices can influence participants’ behavior when completing their movement task, and to be mindful of them not only when designing such experiments, but also when interpreting the ensuing results. But while a tailored set of design choices can have its advantage for individual scientific endeavors, this approach can also have its downside when it comes to replications or meta-analyses (see Elson, 2019).

The Simon effect that we used is a rather basic and robust finding, and here it serves as a proxy for other experimental manipulations in which participants are to make a choice between two spatially separated response options. Because such tasks are routinely used in research that capitalizes on mouse- or finger-tracking, the above-mentioned recommendations might provide a usable guideline when deciding on those design choices. Still, other tasks might require a slightly different design. Also, with the current line of research, we only varied one factor within a setup, neglecting any effects that specific combinations of design choices might come with. We have briefly alluded to some of these interactions in the discussions of the individual experiments, e.g., how to prioritize target distance over spatial layout. Similarly, we discussed how both a hit detection based on a hover/touch criterion and the use of larger target areas individually might be able to reduce omission rates, but a combination of large target areas and such a hit condition could likely lead to overall less consistent movements. So while we were able to derive specific recommendations for each design choice, specific combinations might come with additional pitfalls or benefits.

That said, we found that any of the tested design choices was principally able to detect the Simon effect, so the movement-tracking setup overall seems to be somewhat robust towards these methodological variations. Researchers wanting to employ this methodology therefore only face a low starting hurdle, and with the freely available software or with basic programming skills, they can easily give it a go without exhaustive piloting and testing of the basic paradigm. We hope that the current overview of the methodological parameters involved can lower these starting hurdles even further.

## Footnotes

1 Note that we do not intend to criticize this aspect of the MouseTracker program, as the program is specifically designed to offer out-of-the-box applications for researchers without the necessary programming expertise to create custom setups. This goal necessarily enforces several design choices on its users, so that limitations are inevitable when intending to provide simple but accessible solutions for mouse-tracking research.

2 Although it would be possible to display an image of a circle within the rectangular starting and target areas to make it look like they were round, all DVs would still be computed based on the rectangular borders. This could lead to confusing instances for participants when they click just outside the circle, which still registers as a response.

3 The iTracker produced higher omission rates because it was the only setup where an omission was possible by prematurely lifting the finger from the touchscreen. These trials were terminated after the finger was prematurely lifted and counted as an omission, as no continuous movement trajectory to one of the target areas could be recorded. See also Experiment 4 for a means to reduce the omission rates.

4 Omission errors in the MouseTracker describe those cases where participants clicked on the edge of the square target area that was not contained in the virtual circular target area that we used during preprocessing. Therefore, they do not constitute omission errors per se; still we excluded these few trials so that the same criteria applied to all input devices.

5 We are aware that there are alternative names and abbreviations in the literature for some of the measures that we describe here. To keep things simple and brief, we refrained from listing specific alternatives.

6 Most measures can also be computed on the raw trajectory data, i.e., prior to time-normalization. Even though both variants will yield highly similar outcomes, time-normalization is necessary to plot average trajectories. Because such plots are routinely provided in empirical reports, we believe it to be useful to compute all relevant statics after rather than before time-normalization.

7 We used the multivariate approach here, which we believe is preferable with a sufficiently large sample size and a limited number of conditions, because it does not depend on sphericity assumptions, and therefore also does not require any corrections in the case of violations.

8 After the data recording session, some participants reported that in a small fraction of trials, they were shown error feedback in spite of correct responses. This error could not be replicated by the experimenters. We did not remove these participants’ performance data, as feedback was only presented after a movement was completed, but we removed their questionnaire data, as erroneous feedback might possibly taint the impression of one of the setups (e.g., judgment of error robustness). With the complete sample, the effect of overall easiness did not emerge, all other effects did.

9 In addition to yielding the strongest compatibility effects of the three setups, ITs of the iTracker were considerably higher overall than those for the two mouse-operated setups. This pattern likely relates to biophysical differences between finger movements and mouse movements. Interestingly, however, using direct finger movements rather than transformed (mouse) movements might thus induce response strategies that mirror findings from research on expertise: Experts in a given domain tend to preplan more strongly than novices, so that their IT data often yields robust effects (Incera & McLennan, 2016; Jusyte et al., 2017).

10 We chose to base our assessment of the individual distributions on sample kurtosis rather than combined measures of skewness and kurtosis such as the bimodality coefficient, because the latter statistic can be distorted for strongly skewed distributions such as the distribution observed for the iTracker (Knapp, 2007; Pfister, Schwarz, Janczyk, Dale, & Freeman, 2013).

11 A promising intermediate solution would be the use of “mousetrap” (Kieslich & Henninger, 2017), which offers a plugin to integrate mouse-tracking with OpenSesame without needing to program the task manually. This package was released after data collection had been completed for Experiment 1 so that it could not be included in this analysis. For a thorough guide on how to use the tool, see Kieslich, Henninger, Wulff, Haslbeck, & Schulte-Mecklenbeck (2019).

12 As an alternative to tying the stimulus onset to the spatial distance that has been traveled, some studies employ a (variable) dwell time within the starting area before displaying the stimulus (e.g., Bundt, Ruitenberg, Abrahamse, & Notebaert, 2018; Wirth, Dignath, Pfister, Kunde, & Eder, 2016). This does not necessarily require one to postpone the decision process to the execution stage, but a variable dwell time circumvents an automatic movement initiation after a fixed time period, and it further separates the motor component of clicking/touching from the initiation of the movement.

13 We hypothesized that this might be due to the fact that target size was manipulated within participants, and might become more variable if participants had only encountered the large target areas. We tested this hypothesis empirically by analyzing only the first condition of each participant. Still, we found that participants on average seemed to aim for the center of the target area, with final movement positions within the perimeter of the smallest target area for all conditions.
